# Thermal stress effects on grain yield in *Brachypodium distachyon *occur via H2A.Z-nucleosomes

**DOI:** 10.1186/gb-2013-14-6-r65

**Published:** 2013-06-25

**Authors:** Scott A Boden, Monika Kavanová, E Jean Finnegan, Philip A Wigge

**Affiliations:** 1 Department of Cell and Developmental Biology, John Innes Centre, Norwich NR4 7UH, UK; 2 CSIRO Plant Industry, GPO Box 1600, Canberra ACT 2601, Australia; 3 Current address: Lehrstuhl für Grünlandlehre, Technische Universität München, Alte Akademie 12, D-85350, Freising-Weihenstephan, Germany; 4 Current address: Sainsbury Laboratory, Cambridge University, 47 Bateman Street, Cambridge CB2 1LR, UK

## Abstract

**Background:**

Crop plants are highly sensitive to ambient temperature, with a 1 ºC difference in temperature sufficient to affect development and yield. Monocot crop plants are particularly vulnerable to higher temperatures during the reproductive and grain-filling phases. The molecular mechanisms by which temperature influences grain development are, however, unknown. In *Arabidopsis thaliana*, H2A.Z-nucleosomes coordinate transcriptional responses to higher temperature. We therefore investigated whether the effects of high temperature on grain development are mediated by H2A.Z-nucleosomes.

**Results:**

We have analyzed the thermal responses of the Pooid grass, *Brachypodium distachyon*, a model system for crops. We find that H2A.Z-nucleosome occupancy is more responsive to increases in ambient temperature in the reproductive tissue of developing grains compared withvegetative seedlings. This difference correlates with strong phenotypic responses of developing grain to increased temperature, including early maturity and reduced yield. Conversely, temperature has limited impact on the timing of transition from the vegetative to generative stage, with increased temperature unable to substitute for long photoperiod induction of flowering. RNAi silencing of components necessary for H2A.Z-nucleosome deposition is sufficient to phenocopythe effects of warmer temperature on grain development.

**Conclusions:**

H2A.Z-nucleosomes are important in coordinating the sensitivity of temperate grasses to increased temperature during grain development. Perturbing H2A.Z occupancy, through higher temperature or genetically, strongly reduces yield. Thus, we provide a molecular understanding of the pathways through which high temperature impacts on yield. These findings may be useful for breeding crops resilient to thermal stress.

## Background

Members of the Pooideae grass family, including wheat, barley, oat and rye, are a major source of human nutrition. The phenology of these crop plants, and the yield and quality of grain produced are significantly influenced by temperature [1,2], making them vulnerable to climate change [3,4].

The effects of temperature at various stages of cereal development have been extensively studied, and optimal temperatures determined for phenological phases from sowing and emergence through to grain development (reviewed in [5]). During vegetative stages, the effects of temperature on growth are evident by the rise in leaf extension rates that occur as temperature increases [6,7]. During generative stages, the influence of temperature on leaf extension rate increases, suggesting that monocot plants have varying degrees of thermal sensitivity depending on their developmental stage [7]. This is evident during late reproductive stages, where the effects of thermal stress are significantly stronger at anthesis and stages thereafter, compared to the double ridge stage, which is the earliest morphological sign of a reproductive plant [8]. Importantly, this includes a major effect of increasing temperature during endosperm development, with growth at moderately high temperatures of 27°C to 32°C reducing the duration of grain filling without a compensatory increase in the rate of grain filling, resulting in significantly reduced yield [9-12]. Increased temperatures also affect the transcriptome of developing grain, resulting in grain at elevated temperatures having a more advanced developmental age [13-15]. Taken together, these results indicate there is a genome-wide mechanism that integrates thermal information into the transcriptome of developing grain.

In *Arabidopsis thaliana*, H2A.Z-nucleosomes play a key role in mediating the effects of ambient temperature on the transcriptome[16]. H2A.Z-nucleosomes are frequently found at positions surrounding the transcription start site (TSS) [17-22]. Occupancy of H2A.Z-nucleosomes at the TSS restricts access of transcriptional machinery into the gene body, and is reduced as temperature increases [16]. The reduced occupancy occurs irrespective of a given gene's transcriptional response to increased temperature, indicating eviction of H2A.Z is caused by exposure to warmer temperature and not simply a consequence of a higher transcription rate [16]. The developmental phenotypes that occur when *Arabidopsis *plants are exposed to warmer temperatures, including accelerated flowering, are constitutively present at cooler temperatures in genotypes compromised in their ability to incorporate H2A.Z into chromatin [16,23-26]. H2A.Z-nucleosomes therefore provide a genome-wide mechanism by which the transcriptome can be coordinated with temperature to fine-tune development in response to the environment.

To understand how crop plants respond to warmer temperatures we have used *Brachypodium distachyon*, a model Pooid grass and close relative of wheat and barley, which is a good exemplar of cereal biology and grain development [27,28]. We assessed the effects of temperature on plant phenology and H2A.Z-nucleosomes of thermally responsive genes, and find that they are more pronounced in developing grain compared to vegetative seedlings. Grain from transgenic plants deficient in H2A.Z deposition resemble those of wild-type plants grown at higher temperature. Our results suggest that H2A.Z-nucleosomes are responsible for the increased thermal sensitivity of reproductive grain-filling tissue compared to vegetative tissue in monocot crop plants.

## Results

### Warmer ambient temperature is not sufficient to induce flowering in *Brachypodium*

**Figure 1:**
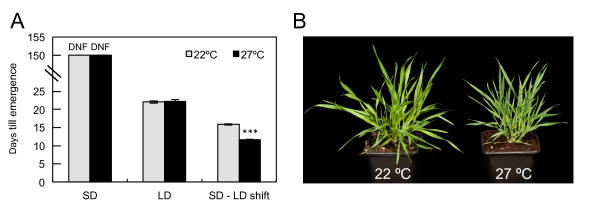
**Higher ambient temperature is not sufficient to induce flowering in *Brachypodium distachyon ***. **(a) **Plants were grown in one of three photoperiod conditions: SD (14 h light/10 h dark), LD (20 h/4 h) or in LD after shifting from SD. In each condition, plants were grown at either 22°C (grey) or 27°C (black). DNF (did not flower) indicates the non-flowering phenotype of plants grown in SD for 150 days. Values are the mean ± standard error of ten plants. (***P < 0.001). **(b) **SD grown plants after 50 days at constant temperature of 22°C and 27°C.

Flowering is a major phase transition in plants. In *A. thaliana*, the floral transition is highly responsive to ambient temperature, with growth at 27°C sufficient to overcome late flowering in short photoperiods [29]. Flowering in natural accessions of *B. distachyon *responds to a shift from long days (LD) to short days (SD) [30]. In our growth conditions, the accession Bd21 did not flower even after 150 days when grown in SD (14 h light/10 h dark) at 22°C, indicating it is a long-day accession. *Brachypodium *also did not flower in SD at 27°C, indicating that, unlike *Arabidopsis*, increased ambient temperatures are unable to substitute for long day induction of flowering (Figure 1). The effect of increased temperature on flowering was further investigated in LD (20 h light/4 h dark), as well as after transfer from SD to LD. No acceleration of flowering was observed at 27°C compared to 22°C when plants were grown constantly in LD, with flowering at both temperatures occurring 22 days after germination (Figure 1a). Previous studies have suggested that temperate grasses are more thermally responsive during reproductive stages than vegetative development [6,7]. To test if this is the case in *Brachypodium*, we assessed the response to increased temperature following floral initiation. Accordingly, plants were grown at 22°C in SD until the emergence of leaf 7, and then transferred to LD for 2 days, which is sufficient for the induction of flowering by transcriptional activation of *Flowering Locus T *(Figure S1 in Additional file 1). Thereafter, plants were either maintained at 22°C or transferred to 27°C, also in LD. By limiting the plant's exposure to warmer temperatures following its commitment to reproductive development, head emergence was found to occur 4 days earlier at 27°C compared to 22°C (Figure 1a). These results indicate that increased temperatures can accelerate flowering in the presence of favorable photoperiods, but are not alone sufficient to trigger flowering.

**Figure 2:**
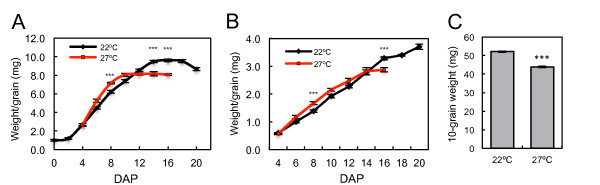
**Increased ambient temperature reduces grain yield in *Brachypodium***. **(a,b) **Fresh weight (a) and dry weight (b) of developing grain from plants grown at either 22/17°C (black line) or 27/22°C (red line) (day/night temperatures). Data are the replicate of 5 replicate plants, including measurements of at least 20 seed. Values are the mean ± standard error (**P < 0.01). **(c) **Final yield measurements in units of weight per 10 grain for plants transferred from 22/17°C to 27/22°C throughout grain-filling, then transferred back to 22/17°C at 16 days after pollination, compared to plants grown constantly at 22/17°C (***P < 0.001). Data are from 20 biological repeats. Values are the mean ± standard error.

This lack of responsiveness of vegetative plants to 27°C could be because *Brachypodium *has evolved to respond to a different range of temperatures than *Arabidopsis*, or because it is differentially responsive to temperature depending on its developmental phase. To assess the latter possibility, we assayed the effects of high temperature on grain development, because it is a late-reproductive stage that is particularly sensitive to thermal stress [2,11]. Plants were grown at 22/17°C day/night cycles until the onset of endosperm development [28], and then maintained at this temperature or shifted to 27/22°C. These temperature treatments were used because growth between 27 and 30°C is recognized as a moderately high thermal stress that adversely affects yield in wheat and barley, compared to control conditions at 20 to 24°C [11,12,15,31]. At 27°C, grain weight initially increased faster than at 22°C, such that after 4 days at 27°C, grain was 14% heavier than at 22°C (*P *< 0.005), indicating an accelerated rate of grain development (Figure 2a,b). However, grain-filling ceased 10 days after pollination (DAP) at 27°C while it continued until 16 DAP at 22°C, which caused a significant reduction in final fresh and dry weights for grain grown at 27°C. The reduced duration of grain-filling was confirmed with mature grain weight measurements that show a 16% decrease (*P *< 0.001) at the higher temperature (Figure 2c). Therefore, grain development is significantly affected by increased ambient temperature, with higher temperatures accelerating the rate of grain development so that there is a reduced developmental window during which grain fill occurs, causing a reduction in yield.

### The *Brachypodium*transcriptome responds to changes in ambient temperature

**Figure 3:**
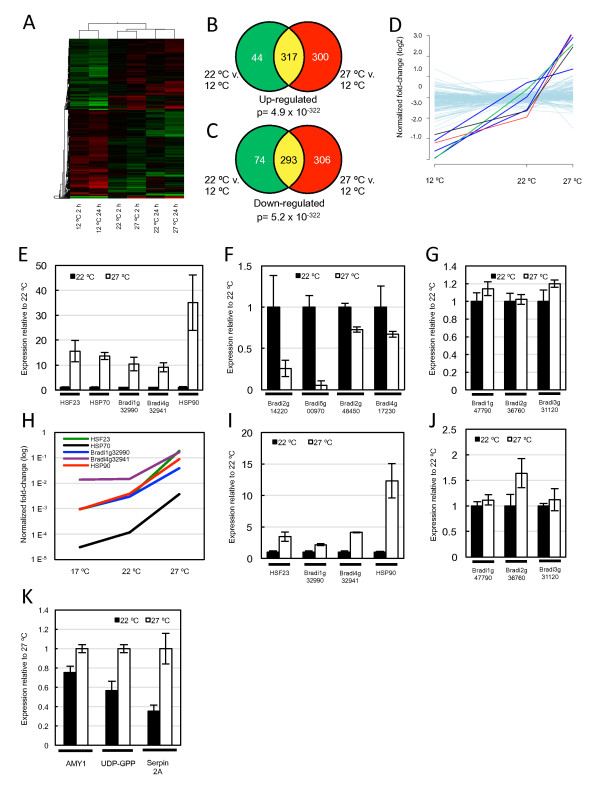
**The *Brachypodium*transcriptome responds to changes in ambient temperature**. **(a) **Transcript profiling experiment shows a robust response to changes in ambient temperature in vegetative seedlings. The heat map depicts all differentially expressed genes (DEGs) with at least two-fold change in any of the temperature treatments as determined from two-way ANOVA (*P *for temperature effect ≤ 0.05). Expression levels of up-regulated genes are in shades of red and of down-regulated genes in shades of green. **(b,c) **Venn diagrams of total numbers of up-regulated (b) or down-regulated (c) DEGs in vegetative seedlings after 24 h shift to either 22°C (green), 27°C (red), or in both temperatures (yellow). The two-tailed *P*-values for the significance of the overlap represented under the Venn diagrams have been calculated using Fisher's exact test. **(d)***HSF23 *(green line), *HSP70 *(black line) and *HSP90 *(red line) are induced strongly with increasing temperature, in contrast to other heat-shock genes (grey) that do not respond significantly over the temperature range assessed. **(e-g) **Quantitative real-time PCR (qRT-PCR) analysis of genes that are up-regulated by increasing temperature (e), down-regulated (f) or show constant expression (g) in vegetative seedlings 24 h after temperature-shift. **(h)**qRT-PCR analysis of up-regulated genes in plants grown constantly at either 17°C, 22°C or 27°C. **(i,j)**qRT-PCR analysis of genes in developing grain that are up-regulated by increasing temperature (i), or remain constant within the temperature range (j). **(k)**qRT-PCR analysis of genes that are up-regulated by temperature with known roles in developing grain. Data are from at least three biological replicates.

Having observed that reproductive tissue appears to be more developmentally responsive to increased temperature than vegetative plants, we sought to test if this is reflected in the regulation of transcription and chromatin state by temperature. To identify marker genes up-regulated in response to increased ambient temperature, we assayed the transcriptional responses of vegetative plants shifted from 12°C to 22°C or 27°C for 2 and 24 hours, using whole genome microarrays and quantitative real-time PCR (qRT-PCR), to obtain rapid and longer-term transcriptomic responses to temperature change. In *Brachypodium *seedlings, 9% of all expressed transcripts were either significantly up- or down-regulated at least two-fold after the shift from 12°C in at least one of the temperature treatments (two-way ANOVA, temperature effect *P *≤ 0.05; Figure 3a-c; Figure S2a-b in Additional file 1). Importantly, as in other systems [32], transfer to 27°C is below the threshold for inducing most heat shock genes, while *HSP70*, *HSF23 *and *HSP90 *are strongly induced (Figure 3d). *Bradi4g32941 *and *Bradi1g32990 *were also selected as genes for further analysis as they too were up-regulated at 27°C (Figure 3e). This induction occurs both in response to short- and medium-term shifts as well as in plants grown continuously at the warmer temperature, suggesting that these genes are responding to absolute temperature, and are not dependent on a temperature change for their expression to be induced (Figure 3e,h). We also identified genes whose transcript levels were down-regulated, such as *Bradi2g14220*, *Bradi5g00970*, *Bradi2g48450 *and *Bradi4g17230 *(Figure 3f), as well as genes that were unaffected by increased temperature, including *Bradi3g31120 *and *Bradi1g47790 *(Figure 3g). These results indicate that while the higher temperature regime does not trigger flowering in short photoperiods, the transcriptome of young seedlings remains thermally responsive.

To determine if this transcriptional response to temperature is tissue specific, we analyzed the expression of these marker genes in developing grain grown at either 22/17°C (day/night) or 27/22°C. We assessed the transcriptional response during endosperm development as this is the stage of grain development in wheat that responds strongly to increased temperature [11,15]. Hence, at 6 to 8 DAP, plants were either shifted to 27/22°C or maintained at 22/17°C, and grains were harvested after 24 h. We found that *HSF23*, *HSP90*, *Bradi4g32941 *and *Bradi1g32990 *were also up-regulated in developing grain (Figure 3i) and that *Bradi3g31120 *and *Bradi1g47790 *transcript levels remained constant between the two temperature treatments (Figure 3j). Expression levels of *HSP70 *were very low and did not appear to be thermally regulated in developing grain, which is similar to results in *Arabidopsis *where *HSP70 *was not temperature-regulated in seeds [33]. We could not detect expression of the genes that weredown-regulated by higher temperature in young seedlings. We also assessed the expression of some genes with important roles during grain development (Figure 3k) shown to be thermally responsive during grain filling in wheat [13], including beta-amylase (*AMY1*) and UDP-glucose pyrophosphorylase (*UDP-GPP*) from the starch metabolism pathway, and *Serpin 2A*, which has a role in plant defense [13]. These genes were up-regulated in grain grown at 27/22°C, relative to 22/17°C, which is consistent with observations in wheat. These results indicate that the developing grain of *Brachypodium *responds transcriptionally to increased ambient temperature, and that some genes display a shared transcriptional response to thermal stress in young seedlings and developing grain.

### H2A.Z-nucleosomes are more thermally responsive in developing grain than vegetative seedlings

**Figure 4:**
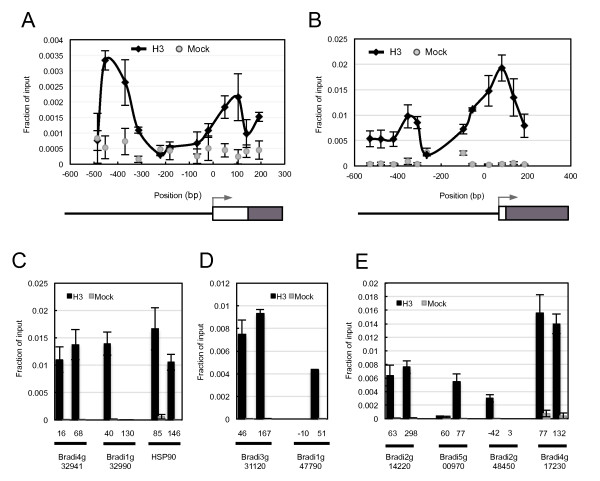
**Nucleosome positioning by H3 ChIP analysis**. **(a,b)**ChIP of cross-linked H3 at the promoter sites of *HSF23 *(a) and *HSP70 *(b) reveal well-positioned -1 and +1 nucleosomes. The x-axis indicates the central position of each amplicon relative to the TSS. In each schematic, the promoter (solid line), 5'UTR (white box), exons (black box) and TSS (arrow) are shown. **(c-e)**ChIP of cross-linked H3 for genes that were detected to be up-regulated (c), constant (d) or down-regulated (e) in response to temperature reveal sites that show strong enrichment of H3 at probable +1 nucleosome sites according to *in silico*sequence analysis (see Materials and methods). The x-axis indicates the central position of each amplicon relative to the TSS. Values from H3 and mock reactions are shown in black and grey, respectively. Values are the mean ± standard error of three biological replicates.

The histone variant H2A.Z has an important role in regulating the ambient temperature transcriptome of *Arabidopsis*, and loss of H2A.Z incorporation into nucleosomes surrounding TSSs promotes thermally sensitive developmental responses [16,26]. We therefore investigated the behavior of H2A.Z-nucleosomes in young seedlings and developing grain to determine if these nucleosomes might explain the altered developmental responsiveness of the two tissue types to increased temperature. Nucleosome positions surrounding the TSS were predicted using software prediction algorithms and confirmed using micrococcal nuclease (MNase) digestion and subsequent histone 3 (H3) chromatin-immunoprecipitation (ChIP) (Figure 4a,b; Figure S3 in Additional file 1). For *HSF23 *and *HSP70*, we used quantitative-PCR (qPCR) with primer pairs tiled across the promoter region surrounding the TSS, which revealed the appearance of two characteristic peaks that are suggestive of -1 and +1 nucleosomes (Figure 4a,b). As the software prediction algorithms accurately predicted nucleosome positions for *HSF23 *and *HSP70*, we used the software to identify the likely +1 nucleosomes of the other thermally responsive genes, which were confirmed by qPCR on DNA isolated from H3 ChIP experiments [34] (Figure 4c-e).

**Figure 5:**
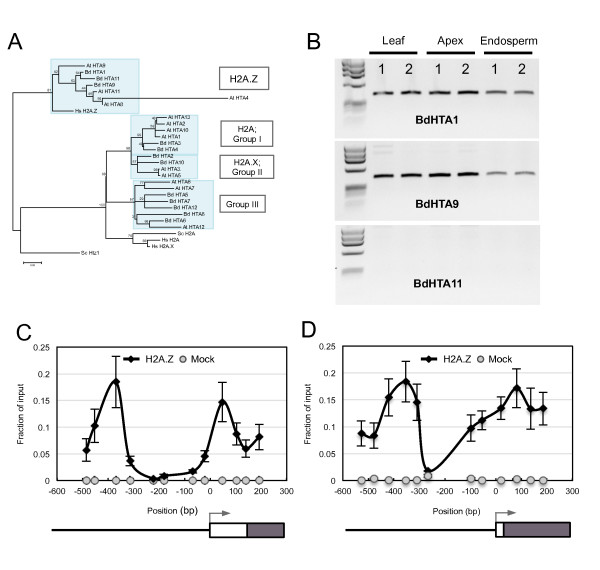
**Identification and nucleosome positioning of *Bd*HTA9 in *Brachypodium***. **(a) **An unrooted maximum-likelihood phylogenetic tree of HTA proteins in *Brachypodium*, *Arabidopsis*, humans and yeast, constructed using MEGA5 with 100 bootstrap replicates, summarizes the evolutionary relationship among the HTA proteins and the separation in four phylogenetic subfamilies. Branches are drawn to scale with scale bar representing the number of substitutions per site. **(b) **Reverse-transcriptase PCR analysis of *BdHTA1*, *BdHTA9 *and *BdHTA11 *from leaf, apex and endosperm tissue of Bd21. Two biological replicates are shown. **(c,d)**ChIP analysis of HTA9:3XFLAG (H2A.Z) at 17°C shows H2A.Z is enriched at the -1 and +1 nucleosomes of *HSF23 *(c) and *HSP70 *(d). The x-axis indicates the central position of each amplicon relative to the TSS. In each schematic, the promoter (solid line), 5'UTR (white box), exons (black box) and TSS (arrow) are shown. Mock reactions (grey) were performed on identical tissue from wild-type plants. Values are the mean ± standard error of three biological replicates.

To investigate if H2A.Z is incorporated into these nucleosomes, we surveyed the *Brachypodium *genome for H2A.Z homologues. Phylogenetic analysis revealed the presence of three H2A.Z genes in *Brachypodium*: *BdHTA1*, *BdHTA9 *and *BdHTA11 *(Figure 5a; Figure S4 in Additional file 1). Transcript analysis in different tissue types, including young seedlings and developing grain, showed that *BdHTA1 *and *BdHTA9 *are strongly expressed, while we could not detect transcripts of *BdHTA11 *(Figure 5b). We therefore selected *BdHTA9*, based on its homology to the H2A.Z orthologue investigated in *Arabidopsis *(*AtHTA11*) and its expression in seedlings and developing grain, for further analysis. To study the dynamics of *BdHTA9 *in response to temperature, we created a *BdHTA9 *3XFLAG form expressed under its native promoter. ChIP analysis using anti-FLAG resin on *Brachypodium *seedlings grown at 17°C revealed the presence of HTA9 at the presumptive -1 and +1 nucleosomes of both *HSF23 *and *HSP70*, and +1 nucleosomes of the other thermally responsive genes (Figure 5c,d). These results are consistent with localization studies of H2A.Z in other organisms, which show incorporation into nucleosomes surrounding the TSS [17-22].

**Figure 6:**
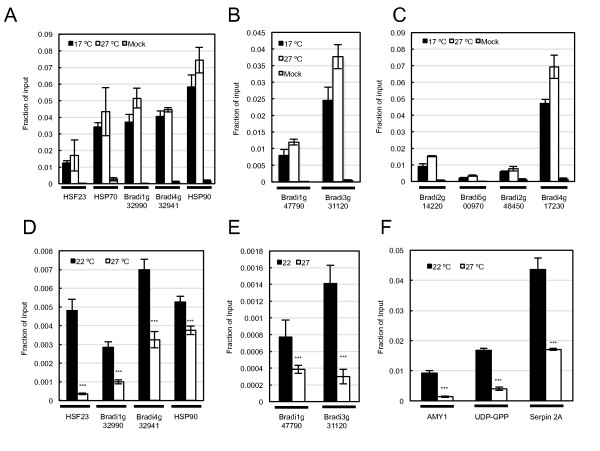
**Occupancy of H2A.Z-nucleosomes is reduced at higher ambient temperatures in developing grain but not in vegetative seedlings**. **(a-c)**ChIP analysis of HTA9:3XFLAG (H2A.Z) at 22°C and 27°C in vegetative seedlings at +1 nucleosomes of genes whose expression was up-regulated (a), remained constant (b) or down-regulated (c) upon an increase in temperature. **(d-f)**ChIP analysis of HTA9:3XFLAG (H2A.Z) at 22/17°C and 22/27°C in developing grain at +1 nucleosomes of genes whose expression was up-regulated (d) in both seedlings and grain, or remained constant (e) upon an increase in temperature. **(f)**ChIP analysis of HTA9:3XFLAG (H2A.Z) at 22/17°C and 27/22°C for genes with roles in grain development. Mock reactions (grey) were performed on identical tissue from wild-type plants. ***P < 0.001.

To determine if eviction of H2A.Z-nucleosomes occurs in vegetative seedlings, as has been described in *Arabidopsis*[16], we performed nucleosome analysis and ChIP for *BdHTA9 *3X FLAG from plants maintained at 17°C or shifted to 27°C. For all genes and nucleosome sites analyzed, we did not detect a decrease in H2A.Z and nucleosome occupancy between 17°C and 27°C (Figure 6a-c; Figure S5 in Additional file 1). This appears to be independent of transcriptional status, since it was observed equally for genes up-regulated, down-regulated or with constant expression after shifting to 27°C (Figure 6a-c).

This apparent stability of H2A.Z-nucleosomes to higher temperature in vegetative plants could be a general feature of chromatin in *Brachypodium*, or it could be an indicator of temperature responsiveness being dependent on developmental phase. To test this latter possibility, we examined the thermal responsiveness of H2A.Z-nucleosomes in developing grain. To ensure that any altered molecular response to temperature was not a consequence of a difference in the physical temperature of the two tissue types, thermal imaging was obtained for both organs in each treatment (Figure S6 in Additional file 1). This analysis showed that there was no difference in physical temperature between vegetative seedlings and developing grain (Figure S6 in Additional file 1). We then investigated H2A.Z-nucleosome behavior under the same conditions used for transcript analysis. *BdHTA9 *3X FLAG plants were grown at 22/17°C until 6 DAP, and then shifted to 27/22°C or maintained at 22/17°C, with grains harvested after 24 h. ChIP and nucleosome analysis showed that there is a striking reduction in HTA9 occupancy at 27/22°C, relative to 22/17°C, for all genes analyzed, including *AMY1*, *UDP-GPP *and *Serpin 2A *(Figure 6d-f). We also observed a comparable reduction in nucleosome levels (Figure S7 in Additional file 1). These results show that in the endosperm of developing grain there is a considerable increase in the mobility of H2A.Z at the +1 nucleosomes of these genes, compared to the same nucleosome sites in vegetative seedlings. In developing grain the transcript levels of *BdHTA9 *did not change with temperature, indicating that the increased mobility of H2A.Z in grain does not occur as a consequence of reduced *BdHTA9 *transcription (Figure S8 in Additional file 1). This result is consistent with the absence of large-scale phenological changes in vegetative plants at 27°C, while a considerable acceleration in grain development is seen at this higher temperature.

### Grain from *ARP6 RNAi *lines phenocopy responses to increased temperature

**Figure 7:**
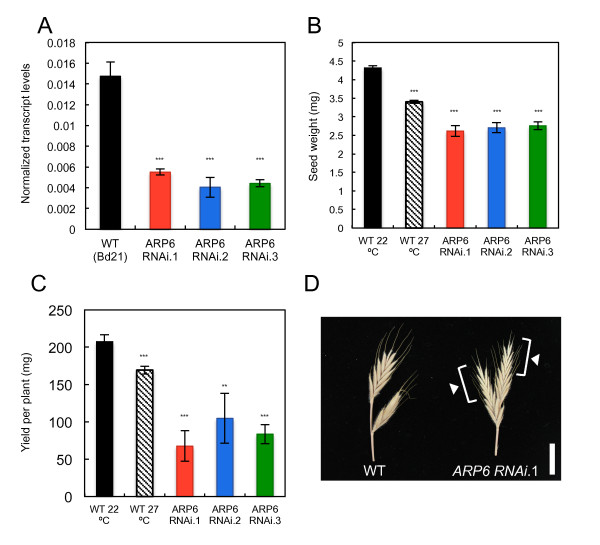
**Seeds from plants with reduced expression of *ARP6 *phenocopy seed from plants grown at higher temperature**. **(a) **Relative expression of *ARP6 *in wild-type (WT; Bd21) and three independent *ARP6 RNAi *transgenic lines (T_1 _generation); ***P < 0.001. **(b,c) **Seed weight measurements (b) and yield per plant (c) in WT (Bd21) at 22/17°C and 27/22°C, as well as three independent transgenic lines at 22/17°C (T_1 _generation). Data are the mean ± standard error of at least 15 grains (**P < 0.01; ***P < 0.001). **(d) **A representative spike from WT Bd21 and *ARP6 RNAi.1 *displaying the empty florets (white arrowheads) that contained aborted grain. Scale bar, 1 cm.

To determine whether loss of H2A.Z-nucleosomes at higher temperature is the cause of altered grain development, or simply a correlated event, we sought to perturb H2A.Z-nucleosome occupancy independently of temperature. H2A.Z is inserted into chromatin through the highly conserved SWR1 complex [24,35,36]. We disrupted the activity of SWR1 using RNA interference (RNAi) silencing of a key conserved component, *ACTIN RELATED PROTEIN 6 *(*ARP6*), of which there is only one copy in the *Brachypodium *genome (*Bradi2g10130*). Using three independent transgenic lines with reduced expression of *ARP6*, we observed a reduction in seed weight (60%, *P *< 0.001) and overall yield per plant (30 to 50%, *P *< 0.01) that was comparable with the reduced yield observed in wild-type plants grown at 27/22°C (Figure 7b,c). We also observed an increased rate of floret sterility and grain abortion in the *ARP6 RNAi *lines (Figure 7c; Table S1 in Additional file 1), which is consistent with observations in wheat where high temperature treatment of early developing grain, prior to the onset of endosperm development, promotes grain abortion and reduction in grain number [37-39]. Other than the phenotypes associated with grain development, the *ARP6 RNAi *lines appeared phenotypically normal and displayed a flowering-time consistent with that of wild-type plants, including an inability to flower in SD (Figure S9 in Additional file 1). These results show that developing grain of *ARP6 RNAi *plants grown at 22/17°C simulate grain harvested from wild-type plants grown at 27/22°C.

**Figure 8:**
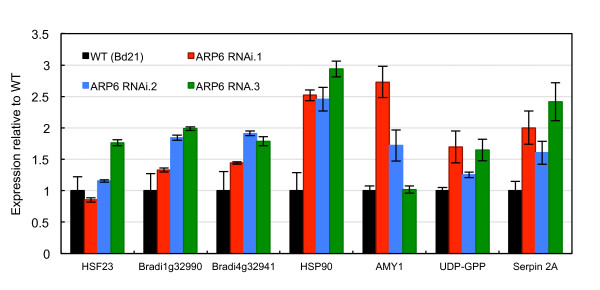
**Genes up-regulated by increased ambient temperature are up-regulated in seeds of *ARP6 RNAi *transgenic lines at 22°C**. Expression of genes that are up-regulated in developing grain by transfer from 22/17°C to 27/22°C (Figure 3) are up-regulated in grain of three *ARP6 RNAi *transgenic lines (T_1 _generation) grown at 22/17°C. Values are the mean ± standard error of 4 replicates, each containing 6 grain at 8 DAP.

To determine if the grain development phenotypes observed in *ARP6 RNAi *plants may be caused by the mis-regulated expression of thermally responsive genes, we compared the transcript levels of thermally induced genes in wild-type and transgenic plants grown at 22/17°C. We used qRT-PCR to assess the relative expression of genes that were up-regulated in developing grain upon transition from 22/17°C to 27/22°C. These results show that reduced activity of *ARP6 *causes elevated transcript levels for genes that were induced by increased temperature in wild-type plants (Figure 8). Some variation in the behavior of transcript levels between RNAi lines may reflect residual *ARP6 *activity. These results indicate that when measured transcriptionally, the developing grain of *ARP6 RNAi *lines grown at 22/17°C resemble those of wild-type plants grown at warmer temperatures.

## Discussion

Ambient temperature has a large effect on plant development, reflected in changes to the transcriptome[32,40]. In *A. thaliana*, H2A.Z-containing nucleosomes are necessary to correctly express the temperature transcriptome[16]. We therefore sought to determine whether H2A.Z-mediated perception of ambient temperature is conserved in monocot plants, and whether the response to increased temperature varies between developing grain and vegetative seedlings.

We have observed that chromatin sites containing H2A.Z-nucleosomes are more responsive to increases in ambient temperature in developing grains than vegetative seedlings. These molecular observations support phenological changes reported in this and other studies, which show that increased temperatures cause a greater response in plants progressing through late-reproductive development compared to those of vegetative stages. For example, the base temperature used to calculate the rate of development using thermal time in wheat is not constant throughout plant development, with the base temperatures for terminal spikelet to anthesis, and anthesis to maturity being considerably higher than that of the vegetative stages [41-43]. A differential response of vegetative and reproductive tissue to moderate and high temperature has also been observed in other plants, such as orchids and strawberry [44,45]. Similarly, it has been shown in *Arabidopsis *that cold temperature treatment causes approximately ten-fold more genes to be induced in seeds than in seedlings [33]. One hypothesis for the distinct responses of vegetative and reproductive tissues is that it reflects an adaptation to low temperatures during early developmental stages and to warmer conditions during reproductive stages [42,43]. Our findings suggest that plants may be able to modulate responsiveness to temperature in a tissue- and lifecycle-dependent manner by controlling the stability of H2A.Z-nucleosomes. To determine how direct the link between H2A.Z-nucleosomes is to the control of grain development in response to temperature, it will be necessary to identify the loci regulating this process and the binding dynamics of H2A.Z-nucleosomes to their promoters.

The effect of temperature on floral initiation has been an active area of research in plant development. Here we find that increased temperature alone cannot promote flowering in non-inductive photoperiods, indicating that *Brachypodium *is an obligate long-day plant and that increased ambient temperature cannot compensate for photoperiod as a floral inductive signal. These observations are consistent with other studies, where it has been shown in wheat and barley that increased temperature in SD conditions does not promote floral initiation, but in fact delays the onset of reproductive development [46,47]. Similarly, in strawberry, olive, rice flowers, perennial grasses and orchids moderately high temperatures delay or completely inhibit floral induction [44,45,48-50]. Taken together, these observations suggest that some plants, including annual temperate grasses such as *Brachypodium*, have adopted different strategies to respond to increased ambient temperature compared to *Arabidopsis*, where flowering is accelerated by high temperature.

The results presented in this study indicate that the transcriptional responses of ripening grain to increased temperature, and the accompanied accelerated rate of development and reduction in grain weight, are at least partially mediated by H2A.Z-nucleosomes. Recent studies in wheat and barley have investigated the transcriptional events initiated by moderate and high increases in temperature during grain development [13-15]. These studies have shown that the transcriptome of a developing grain is highly responsive to increased ambient temperatures [14] and that the developmental age of the grain is more advanced at warmer temperatures, as measured transcriptionally [15]. Therefore, it may be possible to reduce the negative effects of thermal stress on grain weight and quality by nullifying the transcriptional response of developing grain to increasing temperatures. This is a concept that has already been investigated in rice, where attenuating the transcriptional activity of α-amylases, which are normally up-regulated by increased temperatures, has been used to ameliorate the deleterious effects that high temperatures have on grain quality [51]. Given the broad role for H2A.Z-nucleosomes in regulating the ambient temperature transcriptome in *Arabidopsis *seedlings, it is possible that a large proportion of the transcriptional responses initiated by increased temperature in developing grain are coordinated by H2A.Z-nucleosomes. Therefore, reducing the responsiveness of H2A.Z-containing nucleosomes in developing grain may help improve yield and grain quality for plants grown in high temperatures. This hypothesis is supported by findings in *Arabidopsis *that plants which lack H2A.Z have fertility defects [26], as well as the results of this study that decreased activity of *ARP6 *causes reduced seed weight and increased expression of thermally responsive genes in plants grown at lower temperatures.

A key question for future research will be to understand the mechanism underlying the differential response of H2A.Z-nucleosomes to increased temperature in reproductive and vegetative tissue. Histone modification is one possible explanation for the more pronounced effect of thermal stress on H2A.Z behavior in nucleosomes of developing grain compared to vegetative seedlings. Recent results in humans have shown that while H2A.Z is localized at sites surrounding the TSS of both active and poised gene promoters, acetylated H2A.Z is only found at TSSs of active genes [52]. In yeast, acetylation of H2A.Z was found to correlate with genome-wide gene activity [53], and confer with nucleosome destabilization and an open conformation of chromatin [54]. It is possible, therefore, that differences in the degree of H2A.Z acetylation may account for tissue-specific variation in thermal responsiveness. In addition to the H2A.Z-nucleosome dependent regulation of transcription, we also observe warm temperature induction of gene expression in seedlings, where no significant decreases in H2A.Z-nucleosomes are seen. This is consistent with work in *Arabidopsis *showing that H2A.Z-nucleosomes do not account for all the transcriptional responses to warmer temperature [16]. The identification of these parallel temperature responsive pathways will be of interest.

The results presented in this work, as well as many phenological studies, suggest that *Brachypodium *and other grasses are more responsive to warmer temperatures as they enter the generative phase of their lifecycle. This may be because the initiation of flowering represents a point of no return, since the reproductive program must be completed. Higher temperatures would then represent an environmental cue associated with the likely onset of temperature stress and drought. Accelerating the developmental transition through this vulnerable stage to the resilient desiccated grain state may therefore be an adaptive response to anticipated abiotic stress. Conversely, activating flowering in response to warmer temperatures independently of photoperiod would risk exposing the inflorescence and the developing grains to frost [55]. This study highlights the value of complementing work in *Arabidopsis *with monocot systems to assess the direct applicability of *Arabidopsis *research to other systems, especially for traits that are important for crop yield, such as temperature perception.

## Conclusions

In common with other monocot plants, *Brachypodium *displays a marked difference in temperature responsiveness depending on the developmental stage. We show that the process of grain-fill is particularly sensitive to warmer temperatures. Our data indicate that H2A.Z-nucleosomes are necessary for coordinating the sensitivity of *Brachypodium *to increased temperature during grain development. Perturbing H2A.Z occupancy, through higher temperature or genetically, strongly reduces yield. Thus, we provide a molecular understanding of the pathways through which high temperature impacts on yield. These findings may be useful for breeding crops resilient to thermal stress.

## Materials and methods

### Plant material and growth conditions

All experiments were carried out using *Brachypodium distachyon *. Material included wild-type Bd21 and transformed lines containing BdHTA9 with a carboxy-terminal 3X FLAG tag, including native promoter with all exons and introns, and transformed lines containing a RNAi construct containing sequence complementary to *ACTIN RELATED PROTEIN 6 *(*ARP6*) (nucleotides 360 to 508 of open reading frame). Transformation was performed as described in [56]. All plants were grown in growth cabinets (Microclima MC1000E, Snijders Scientific, Tilburg, Netherlands) with 70% relative air humidity and 350 µmol m^-2 ^s^-1 ^PPFD (Photosynthetic Photon Flux Density).

For flowering-time experiments, plants were grown in one of three photoperiod regimes: i) SD (14 h light/10 h dark); ii) LD (20 h/4 h); or iii) transferred from SD to LD at the seven leaf stage. Plants were grown at constant temperatures of either 22°C or 27°C. For analysis of temperature effects on grain development, plants were grown in SD at 22/17°C (day/night temperatures) until the seven-leaf stage to synchronize development, and shifted to LD at the same temperature. After head emergence, date of pollination was monitored for the main stem. At 5 DAP, plants were either maintained at 22/17°C or shifted to 27/22°C and developing grains were collected at two-day intervals. At 16 DAP, plants grown at 27/22°C were shifted back to 22/17°C until maturity to measure final yield. Dry weight of developing grain was measured after desiccating grain at 60°C for 10 days. For each time point and temperature, a total of 20 seed were measured that were collected from 5 plants.

For transcript analysis of vegetative plants, seedlings were grown at 17°C in SD until emergence of the third leaf, and transferred to 12°C for 48 h before shifting to either 22°C or 27°C. Samples were collected at 2 h and 24 h post-shift. Transcript analysis of developing grain was performed only on grain harvested from the main stem. Grain were collected from plants grown at 22/17°C until the end of 6 DAP, which were then transferred to 27/22°C or maintained at 22/17°C. Samples were collected 24 h after shift. Each replicate contained pools of 6 grain, from which the glumes had been removed.

### Transcript analysis

Transcript analysis was performed on total RNA extracted using the Plant RNeasy Plant Mini kit (Qiagen, Crawley, West Sussex, UK). Single-stranded cDNA was amplified for microarray analysis using the Ambion WT Expression Kit (Ambion Life Technologies, Paisley, Renfrewshire, UK), labeled and hybridized to the custom *Brachypodium *microarray (Affymetrix, Santa Clara, California, USA) using the GeneChip WT terminal labeling and hybridization kit (Affymetrix). Microarray data were analyzed using GeneSpringGX v.11 (Agilent, Santa Clara, California, USA). For each time-point after the temperature shift (2 h and 24 h) and temperature treatment (12°C, 22°C and 27°C), two to three biological replicates were analyzed. Samples taken before the shift at 12°C 0 h were considered to be equivalent to the 12°C 24 h treatment for the purpose of the statistical analysis. Raw data were normalized using the RMA algorithm and filtered by expression level using the cut-off value of ≥20 in at least one of the treatments to define the group of genes expressed in vegetative seedlings. The statistical significance of changes in the transcripts with expression levels above the cut-off level was assessed using two-way ANOVA with temperature and time as main factors. The *P-*values were adjusted for multiple testing by the Benjamini and Hochberg's method at a false discovery rate of ≤0.05. The transcripts significantly affected by temperature as the main factor (corrected *P *≤ 0.05) and displaying ≥2-fold change in any of the temperature-time combinations were reported as differentially expressed genes (DEGs). Hierarchical clustering and heat mapping were used to visually display DEGs. The significance of the overlap between the lists of DEGs in different temperature-time treatments represented in Venn diagrams were tested using Fisher's exact test. The DEGs were annotated using the bradinet platform of web tools [57], and are provided as a list in Table S5 in Additional file 2). The data for these experiments have been deposited in ArrayExpress (E-MEXP-3918).

For transcript analysis by qRT-PCR, cDNA was synthesized using the Fermentas First Strand cDNA synthesis kit (Thermo Scientific, St. Leon-Rot, Germany). qRT-PCR was performed using SYBR Green I (Roche, Burgess Hill, West Sussex, UK) and a Roche LightCycler 480. All qRT-PCR data points are the average of three biological replicates, with two technical replicates performed in each reaction. See Table S2 in Additional file 3 for oligonucleotide sequences used for qRT-PCR. Expression of candidate genes was normalized against *SamDC *and *UBC18*[58].

### Nucleosome positioning and ChIP

Nucleosome positioning and ChIP were performed essentially as described [16], with minor modifications. For positioning of nucleosomes in vegetative plants, chromatin from seedlings grown at 17°C was cross-linked using 1% formaldehyde. Chromatin was fragmented using 0.2 units of micrococcal nuclease (Sigma, Gillingham, Dorset, UK) as described [16]. Nucleosome positions were identified by immunopurification using the H3 antibody (Abcam, ab1791, Cambridge, Cambridgeshire, UK)). Relative enrichment of associated DNA fragments was analyzed by qPCR. Nucleosome occupancy was determined as a fraction of uncut chromatin DNA, with data plotted against the *HSF23 *and *HSP70 *promoters. *HSP70 *was selected for detailed nucleosome positioning because it is the homologous gene used for transcript and H2A.Z-nucleosome analysis in *A. thaliana*[16], and *HSF23 *was selected because it is expressed at a level proportionate to ambient temperature within the range 12 to 27°C. For *in silico *predictions of +1 nucleosome sites, 1 kb and 250 bp of sequence upstream and downstream of the TSS, respectively, were used to query the online nucleosome position prediction software (version 3.0) on the Segal lab website [34]. The two most probable sites were selected for qPCR analysis of H3 ChIP DNA, with amplicons that provided the strongest signal used for further analysis. The center of the amplicon indicates its position relative to the TSS. Oligonucleotide sequences are provided in Table S3 in Additional file 4.

Similar methods as outlined above were used to determine nucleosome and H2A.Z dynamics at different temperatures. Nucleosome and H2A.Z dynamics were assessed using chromatin isolated from seedlings that were grown at 17°C until 10 days after germination, and then either maintained at 17°C or shifted to 27°C for 24 h. Chromatin was digested with MNase, and for nucleosome analysis, mononucleosome-sized fragments were gel purified and used in qPCR. For H2A.Z dynamics, ChIP was performed on MNase digested chromatin using the ANTI-FLAG M2 Affinity Gel (Sigma), and histone-DNA complexes eluted using the 3X Flag peptide (Sigma). For assays of nucleosome and H2A.Z occupancy in developing grain, plants were grown at 22/17°C until 6 DAP. Plants were then either maintained at 22/17ºC or shifted to 27/22°C and developing grains (1 g per ChIP assay) were collected after 24 h (7 DAP), with grain harvested only from the main stem. Native ChIP assays were performed as described above, with the following modifications. Ground tissue was washed twice in extraction buffer I, and three times in extraction buffer II to remove metabolic contaminants. Following MNase digestion, two rounds of centrifugation were performed on uncut chromatin DNA and digested chromatin DNA prior to reverse cross-linking and immunopurification, respectively, to further remove contaminants. Nucleosome and H2A.Z occupancy was determined as a fraction of input DNA that was diluted ten-fold for qRT-PCR. Oligonucleotide sequences are provided in Table S3 in Additional file 4. All ChIP assays were performed at least three times, and two technical replicates were performed for each qRT-PCR.

### Sequence alignment and phylogenetic analysis

*Brachypodium *HTA sequences were identified by BLAST search [59] from public databases using *Arabidopsis *HTA protein sequences as bait (Table S4 in Additional file 5). Multiple sequence alignment was performed using webPRANK[60], and manually corrected using GENEDOC [61]. Phylogenetic trees were constructed in MEGA5 [62] using the maximum likelihood algorithm with the following parameters: JTT substitution model, Gamma distributed rates and partial deletions. Bootstrap values are based on 100 replicates for testing the significance of the nodes.

### Seed weight measurements from *ARP6 RNAi *lines

For wild-type (Bd21) and each transgenic *ARP6 RNAi *line, seed weight measurements were determined for 15 seeds harvested from 3 plants (5 seeds from each replicate plant). Seeds were harvested from basal florets of spikelets from the main spike, and the lemma and palea were removed before weighing. For yield per plant measurements, all seeds were harvested from three replicate plants.

### Statistical analysis

When not described specifically, differences between treatments were tested by Student's *t*-test. Results in figures are shown as means ± standard error.

## Abbreviations

bp: base pair; ChIP: chromatin-immunoprecipitation; DAP: days after pollination;H2A.Z: histone 2A variant Z; H3: histone 3; LD: long day; MNase: micrococcal nuclease; qPCR: quantitative-PCR; qRT-PCR: quantitative real-time PCR; RNAi: RNA interference; SD: short day; TSS: transcription start site; UTR: untranslated region.

## Competing interests

The authors declare that they have no competing interests.

## Authors' contributions

SAB, MK, JF and PAW participated in the design of the study and drafted the manuscript. SAB and JF performed the molecular experiments and plant phenotype analysis. MK performed the statistical analysis of microarray data, sequence alignments and constructed the phylogenetic tree. SAB and PAW conceived the project. All authors read and approved the final manuscript.

## Supplementary Material

Additional file 1 **Supplementary figures**. Click here for file

Additional data file 2 **Table S5**. The differentially expressed genes (DEGs), defined as significantly affected by temperature as the main factor (*P *≤ 0.05) and displaying ≥2-fold change in at least one of the temperature-time treatments. Click here for file

Additional file 3 **Table S2**. Click here for file

Additional file 4 **Table S3**. Click here for file

Additional file 5 **Table S4**. Click here for file

Additional file 6 **Supplementary methods and figure legends**. Click here for file

## References

[B1] Wardlaw IF Dawson IA The tolerance of wheat to high temperatures during reproductive growth. II Grain development. Aust J Agric Res 1989 14 15 24 10.1071/AR9890015

[B2] Wallwork MAB Jenner CF Logue SJ Sedgley M Effect of high temperature during grain-filling on the structure of developing and malted barley grains. Ann Bot 1998 14 587 599 10.1006/anbo.1998.0721

[B3] Battisti DS Naylor RL Historical warnings of future food insecurity with unprecedented seasonal heat. Science 2009 14 240 244 10.1126/science.1164363 19131626

[B4] Lobell DB Schlenker W Costa-Roberts J Climate trends and global crop production since 1980. Science 2011 14 616 620 10.1126/science.1204531 21551030

[B5] Porter JR Gawith M Temperatures and the growth and development of wheat: a review. Eur J Agronomy 1999 14 23 36 10.1016/S1161-0301(98)00047-1

[B6] Peacock JM Temperature and leaf growth in *Lolium perenne *III. Factors affecting seasonal differences. J Appl Ecol 1975 14 685 697 10.2307/2402182

[B7] Parsons AJ Robson JM Seasonal changes in the physiology of S24 perennial rygrass (*Lolium perenne L*.). 1. Response of leaf extension to temperature during the transition from vegetative to reproductive growth. Ann Bot 1980 14 435 444

[B8] Wollenweber R Porter JR Schellberg J Lack of interaction between extreme high temperature events at vegetative and reproductive growth stages in wheat. J Agron Crop Sci 2003 14 142 150 10.1046/j.1439-037X.2003.00025.x

[B9] Tashiro T Wardlaw IF A comparison of the effect of high temperature on grain development in wheat and rice. Ann Bot 1989 14 59 65

[B10] Wardlaw IF Moncur L Patrick JW The response of wheat to high temperature following anthesis. II. Sucrose accumulation and metabolism by isolated kernels. Aust J Plant Physiol 1995 14 399 407 10.1071/PP9950399

[B11] Gooding MJ Ellis RH Shewry PR Schofield JD Effects of restricted water availability and increased temperature on the grain filling, drying and quality of winter wheat. J Cereal Sci 2003 14 295 309 10.1006/jcrs.2002.0501

[B12] Wardlaw IF Moncur I The response of wheat to high temperature following anthesis. I. The rate and duration of kernal filling. Aust J Plant Physiol 1995 14 391 397 10.1071/PP9950391

[B13] Altenbach SB Kothari KM Transcript profiles of genes expressed in endosperm tissue are altered by high temperature during wheat grain development. J Cereal Sci 2004 14 115 126 10.1016/j.jcs.2004.05.004

[B14] Mangelsen E Killian J Harter K Jansson C Wanke D Sundberg E Transcriptome analysis of high-temperature stress in developing barley caryopses: Early stress responses and effects on storage compound biosynthesis. Mol Plant 2011 14 97 115 10.1093/mp/ssq058 20924027

[B15] Wan Y Poole RL Huttly AK Toscano-Underwood C Feeney K Welham S Gooding MJ Mills C Edwards KJ Shewry PR Mitchell RA Transcriptome analysis of grain development in hexaploid wheat. BMC Genomics 2008 14 121 10.1186/1471-2164-9-121 18325108 PMC2292175

[B16] Kumar SV Wigge PA H2A.Z-containing nucleosomes mediate the thermosensory response in Arabidopsis. Cell 2010 14 136 147 10.1016/j.cell.2009.11.006 20079334

[B17] Creyghton MP Markoulaki S Levine SS Hanna J Lodato MA Sha K Young RA Jaenisch R Boyer LA H2A.Z is enriched at polycomb complex target genes in ES cells and is necessary for lineage commitment. Cell 2008 14 649 661 10.1016/j.cell.2008.09.056 18992931 PMC2853257

[B18] Mavrich TN Ioshikhes IP Xiaoyong L Jiang C Venters BJ Zanton SJ Tomsho LP Glaser RL Schuster SC Gilmour DS Istvan A Pugh BF Nucleosome organization in the *Drosophila *genome. Nature 2008 14 358 362 10.1038/nature06929 18408708 PMC2735122

[B19] Raisner RM Hartley PD Meneghini MD Bao MZ Liu CL Schreiber SL Rando OJ Madhani HD Histone variant H2A.Z marks the 5' Ends of both active and inactive genes in euchromatin. Cell 2005 14 233 248 10.1016/j.cell.2005.10.002 16239142 PMC2039754

[B20] Whittle CM McClinic KN Ercan S Zhang X Green RD Kelly WG Lieb JD The genomic distribution and function of histone variant HTZ-1 during *C. elegans *embryogenesis. PLoS Genet 2008 14 e1000187 10.1371/journal.pgen.1000187 18787694 PMC2522285

[B21] Zhang H Roberts DN Cairns BR Genome-wide dynamics of Htz1, a histone H2A variant that poises repressed/basal promoters for activation through histone loss. Cell 2005 14 219 231 10.1016/j.cell.2005.08.036 16239141 PMC2788555

[B22] Zilberman D Coleman-Derr D Ballinger T Henikoff S Histone H2A.Z and DNA methylation are mutually antagonistic chromatin marks. Nature 2008 14 125 129 10.1038/nature07324 18815594 PMC2877514

[B23] Martin-Trillo M *EARLY IN SHORT DAYS 1 (ESD1) *encodes *ACTIN-RELATED PROTEIN 6 *(*AtARP6*), a putative component of chromatin remodelling complexes that positively regulates FLC accumulation in Arabidopsis. Development 2006 14 1241 1252 10.1242/dev.02301 16495307

[B24] Deal RB Topp CN McKinney EC Meagher RB Repression of flowering in Arabidopsis requires activation of *FLOWERING LOCUS C *Expression by the histone variant H2A.Z. Plant Cell 2007 14 74 83 10.1105/tpc.106.048447 17220196 PMC1820970

[B25] March-Díaz R García-Domínguez M Lozano-Juste J León J Florencio FJ Reyes JC Histone H2A.Z and homologues of components of the SWR1 complex are required to control immunity in Arabidopsis. Plant J 2007 14 475 487 10.1111/j.1365-313X.2007.03361.x 17988222

[B26] Coleman-Derr D Zilberman D Deposition of histone variant H2A.Z within gene bodies regulates responsive genes. PLoS Genet 2012 14 e1002988 10.1371/journal.pgen.1002988 23071449 PMC3469445

[B27] Opanowicz M Vain P Draper J Parker D Doonan JH *Brachypodium distachyon*: making hay with a wild grass. Trends Plant Sci 2008 14 172 177 10.1016/j.tplants.2008.01.007 18343709

[B28] Opanowicz M Hands P Betts D Parker ML Toole GA Mills ENC Doonan JH Drea S Endosperm development in *Brachypodium distachyon*. J Exp Bot 2010 14 735 748 21071680 10.1093/jxb/erq309 PMC3003816

[B29] Balasubramanian S Sureshkumar S Lempe J Weigel D Potent induction of *Arabidopsis thaliana *flowering by elevated growth temperature. PLoS Genet 2006 14 e106 10.1371/journal.pgen.0020106 16839183 PMC1487179

[B30] Schwartz CJ Doyle MR Manzaneda AJ Rey PJ Mitchell-Olds T Amasino RM Natural variation of flowering time and vernalization responsiveness in *Brachypodium distachyon*. Bioenerg Res 2010 14 38 46 10.1007/s12155-009-9069-3

[B31] Wardlaw IF Interaction between drought and chronic high temperature during kernel filling in wheat in a controlled environment. Ann Bot 2002 14 469 476 10.1093/aob/mcf219 12324270 PMC4240385

[B32] Samach A Wigge PA Ambient temperature perception in plants. Curr Opin Plant Biol 2005 14 483 486 10.1016/j.pbi.2005.07.011 16054430

[B33] Kendall SL Hellwege A Marriot P Whalley C Graham IA Penfield S Induction of dormancy in Arabidopsis summer annuals requires parallel regulation of DOG1 and hormone metabolism by low temperature and CBF transcription factors. Plant Cell 2011 14 2568 2580 10.1105/tpc.111.087643 21803937 PMC3226211

[B34] Kaplan N Moore IK Fondufe-Mittendorf Y Gossett AJ Tillo D Field Y LeProust EM Hughes TR Lieb JD Widom J Segal E The DNA-encoded nucleosome organization of a eukaryotic genome. Nature 2009 14 362 366 10.1038/nature07667 19092803 PMC2658732

[B35] Krogan NJ Keogh M-C Datta N Sawa C Ryan OW Ding H Haw RA Pootoolal J Tong A Canadien V Rischards DP Wu X Emili A Hughes TR Buratowski S Greenblatt JF A Snf2 family ATPase complex required for recruitment of the histone H2A variant Htz1. Mol Cell 2003 14 1565 1576 10.1016/S1097-2765(03)00497-0 14690608

[B36] Mizuguchi G Shen X Landry J Wu W-H Sen S Wu C ATP-driven exchange of histone H2AZ variant catalyzed by SWR1 chromatin remodeling complex. Science 2004 14 343 348 10.1126/science.1090701 14645854

[B37] Gibson LR Paulsen GM Yield components of wheat grown under high temperature stress during reproductive growth. Crop Sci 1999 14 1841 1846 10.2135/cropsci1999.3961841x

[B38] Tashiro T Wardlaw IF The response to high temperature shock and humidity changes prior to and during the early stages of grain development in wheat. Aust J Plant Physiol 1990 14 551 561 10.1071/PP9900551

[B39] Bhullar SS Jenner CF Responses to brief periods of elevated temperature in ears and grains of wheat. Aust J Plant Physiol 1983 14 549 560 10.1071/PP9830549

[B40] McClung CR Davis SJ Ambient thermometers in plants: From physiological outputs towards mechanisms of thermal sensing. Curr Biol 2010 14 R1086 R1092 10.1016/j.cub.2010.10.035 21172632

[B41] Del Pozo AH García-Huidobro J Novoa R Villaseca S Relationship of base temperature to development of spring wheat. Exp Agriculture 1987 14 21 30 10.1017/S0014479700001095

[B42] Slafer GA Savin R Developmental base temperature in different phenological phases of wheat (*Triticum aestivum*). J Exp Bot 1991 14 1077 1082 10.1093/jxb/42.8.1077

[B43] Angus JF MacKenzie DH Morton R Schafer CA Phasic development in field crops II. Thermal and photoperiodic responses of spring wheat. Field Crops Res 1981 14 269 283

[B44] Heide OM Photoperiod and temperature interactions in growth and flowering of Strawberry. Physiologia Plantarum 1977 14 21 26 10.1111/j.1399-3054.1977.tb01486.x

[B45] Newton LA Runkle ES High-temperature inhibition of flowering of *Phalaenopsis *and *Doritaenopsis *orchids. HortScience 2009 14 1271 1276

[B46] Rawson HM Richards RA Effects of high temperature and photoperiod on floral development in wheat isolines differing in vernalisation and photoperiod genes. Field Crops Res 1993 14 181 192 10.1016/0378-4290(93)90030-Q

[B47] Hemming M Walford SA Fieg S Dennis ES Trevaskis B Identification of high temperature responsive genes in cereals. Plant Physiol 2012 14 1439 50 10.1104/pp.111.192013 22279145 PMC3291267

[B48] Hackett WP Hartmann HT The influence of temperature on floral initiation in the olive. Physiologia Plantarum 1967 14 430 436 10.1111/j.1399-3054.1967.tb07183.x

[B49] Ofir M Kigel J Opposite effects of daylength and temperature on flowering and summer dormancy of *Poa bulbosa*. Ann Bot 2006 14 659 666 10.1093/aob/mcl021 16467351 PMC2803668

[B50] Halevy AH Shlomo E Environmental factors affecting flowering of rice flower (*Ozothamnus diosmifolius*, Vent.). Scientia Horticulturae 2001 14 303 309 10.1016/S0304-4238(00)00182-5

[B51] Hakata M Kuroda M Miyashita T Yamaguchi T Kojima M Sakakibara H Mitsui T Yamakawa H Suppression of α-amylase genes improves quality of rice grain ripened under high temperature. Plant Biotechnol J 2012 14 1110 1117 10.1111/j.1467-7652.2012.00741.x 22967050

[B52] Valdes-Mora F Song JZ Statham AL Strbenac D Robinson MD Nair SS Patterson KI Tremethick DJ Stirzaker C Clark SJ Acetylation of H2A.Z is a key epigenetics modification associated with gene deregulation and epigenetic remodeling in cancer. Genome Res 2012 14 307 321 10.1101/gr.118919.110 21788347 PMC3266038

[B53] Millar CB Xu F Zhang K Grunstein M Acetylation of H2A.Z Lys 14 is associated with genome-wide gene activity in yeast. Gene Dev 2006 14 711 722 10.1101/gad.1395506 16543223 PMC1413291

[B54] Ishibashi T Dryhurst D Rose KL Shabanowitz J Hunt DF Ausio J Acetylation of vertebrate H2A.Z and its effect on the structure of the nucleosome. Biochemistry 2009 14 5007 5017 10.1021/bi900196c 19385636 PMC2850812

[B55] Marcellos H Single WV Frost injury in wheat ears after ear emergence. Aust J Plant Physiol 1984 14 7 15 10.1071/PP9840007

[B56] Alves SC Worland B Thole V Snape JW Bevan MW Vain P A protocol for *Agrobacterium*-mediated transformation of *Brachypodium distachyon *community standard line Bd21. Nat Protoc 2009 14 638 649 10.1038/nprot.2009.30 19360019

[B57] bradinet. http://aranet.mpimp-golm.mpg.de/bradinet

[B58] Hong S-Y Seo P Yang M-S Xiang F Park C-M Exploring valid reference genes for gene expression studies in *Brachypodium distachyon *by real-time PCR. BMC Plant Biol 2008 14 112 10.1186/1471-2229-8-112 18992143 PMC2588586

[B59] Altschul S Gish W Miller W Myers E Lipman D Basic Local Alignment Search Tool. J Mol Biol 1990 14 405 410 10.1016/S0022-2836(05)80360-2 2231712

[B60] Löytynoja A Goldman N webPRANK: a phylogeny-aware multiple sequence aligner with interactive alignment browser. BMC Bioinformatics 2010 14 579 10.1186/1471-2105-11-579 21110866 PMC3009689

[B61] Nicholas K Nicholas Jnr H Deerfield D II GeneDoc: Analysis and Visualization of Genetic Variation. EMBNEWNEWS 1997 14 14

[B62] Tamura K Peterson D Peterson N Stecher G Masatoshi N Kumar S MEGA5: Molecular evolutionary genetics analysis using maximum likelihood, evolutionary distance, and maximum parsimony methods. Mol Biol Evol 2011 14 2731 2739 10.1093/molbev/msr121 21546353 PMC3203626

