# Supplementary material for: Thermal stress effects on grain yield in Brachypodium distachyon occur via H2A.Z-nucleosomes
Source: Genome Biol. 2013 Jun 25;14(6):R65. doi: 10.1186/gb-2013-14-6-r65 (PMC4062847; doi:10.1186/gb-2013-14-6-r65)
Supplement: Additional data file 2 — Table S5. The differentially expressed genes (DEGs), defined as significantly affected by temperature as the main factor (P ≤ 0.05) and displaying ≥2-fold change in at least one of the temperature-time treatments. [file gb-2013-14-6-r65-S2.DOCX]

Table S2: Oligonucleotide sequences

Oligonucleotide sequences used in quantitative PCR assays

| Gene | Locus ID | Orientation | Sequence |
| --- | --- | --- | --- |
| HSF23 | Bradi3g58590 | Sense | atcaaggccgagatgaagaac |
|  |  | Anti-sense | tagtcgacgttgacctggaag |
| HSP70 | Bradi2g23250 | Sense | caacaccgtcttcgatgcg |
|  |  | Anti-Sense | gtgctggacgacaatcat |
| HSP90 | Bradi5g02037 | Sense | tctccaactcctcagatgcg |
|  |  | Anti-sense | ggtacgaggcggatgaagag |
| Bradi1g32990 | Bradi1g32990 | Sense | cagtaaagcagacctcagaaaga |
|  |  | Anti-Sense | gctggcaaacatcatcagc |
| Bradi4g32941 | Bradi4g32941 | Sense | gaaagatgagaaagaggactacaag |
|  |  | Anti-sense | gcttctgattcccagtgtcc |
| Bradi2g14220 | Bradi2g14220 | Sense | agctttcagcgaaggagaca |
|  |  | Anti-Sense | acaagcctctcacaggatgc |
| Bradi5g00970 | Bradi5g00970 | Sense | cttcttcgtaaagcgtggc |
|  |  | Anti-sense | ccgttgtcgaggtactcctt |
| Bradi2g48450 | Bradi2g48450 | Sense | ctttcgtcctcgacatcttc |
|  |  | Anti-Sense | ggacttgccaactacctcgt |
| Bradi4g17230 | Bradi4g17230 | Sense | gaagttcaagaggatgtgcg |
|  |  | Anti-sense | ccaccaccacgatgtcct |
| Bradi1g47790 | Bradi1g47790 | Sense | gttggtgttacaatggagataatg |
|  |  | Anti-Sense | ggtccgatctctttctcagg |
| Bradi2g36760 | Bradi2g36760 | Sense | gctttatgagattgctagtcaaag |
|  |  | Anti-sense | tttgctgccgaaatacttga |
| Bradi3g31120 | Bradi3g31120 | Sense | actacctatctcggttgacaca |
|  |  | Anti-Sense | gacaacgagccagttcttcc |
| AMY1 | Bradi1g25440 | Sense | gtccaacaggtgctgagtgc |
|  |  | Anti-sense | accttttggtctcgcatttc |
| UDP-GPP | Bradi4g37350 | Sense | taacttccttgcccgattca |
|  |  | Anti-Sense | gatggtcaccttgcccttg |
| Serpin 2A | Bradi1g14730 | Sense | ccgttttccacaagtcgttt |
|  |  | Anti-Sense | ggattgacctaagcgtgacca |
