# Supplementary material for: Thermal stress effects on grain yield in Brachypodium distachyon occur via H2A.Z-nucleosomes
Source: Genome Biol. 2013 Jun 25;14(6):R65. doi: 10.1186/gb-2013-14-6-r65 (PMC4062847; doi:10.1186/gb-2013-14-6-r65)
Supplement: Additional file 3 — Table S2. [file gb-2013-14-6-r65-S3.PDF]

Figure S1

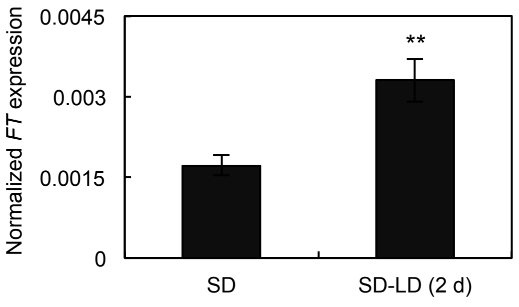

Figure S2

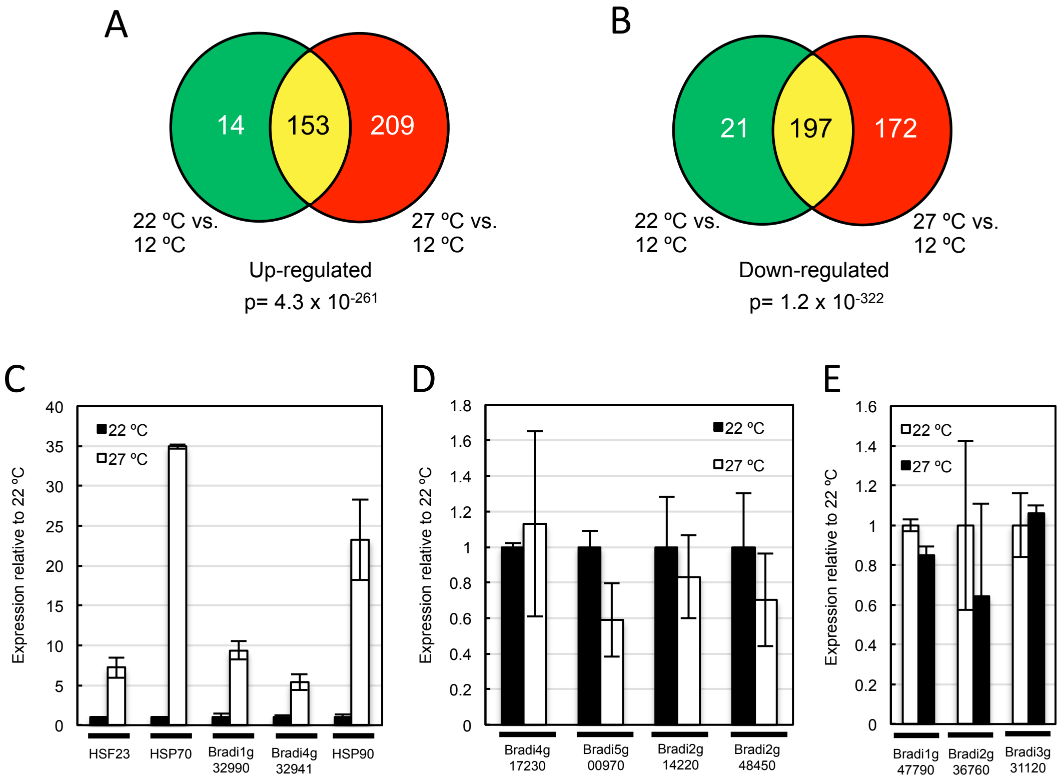

A
14
153
209
22 ºC vs.
12 ºC
27 ºC vs.
12 ºC
Up-regulated
p= 4.3 x 10-261
B
21
197
172
22 ºC vs.
12 ºC
27 ºC vs.
12 ºC
Down-regulated
p= 1.2 x 10-322
C
Expression relative to 22 ºC
22 ºC
27 ºC
HSF23
HSP70
Bradi1g 32990
Bradi4g 32941
HSP90
D
Expression relative to 22 ºC
22 ºC
27 ºC
Bradi4g 17230
Bradi5g 00970
Bradi2g 14220
Bradi2g 48450
E
Expression relative to 22 ºC
22 ºC
27 ºC
Bradi1g 47790
Bradi2g 36760
Bradi3g 31120

Figure S3

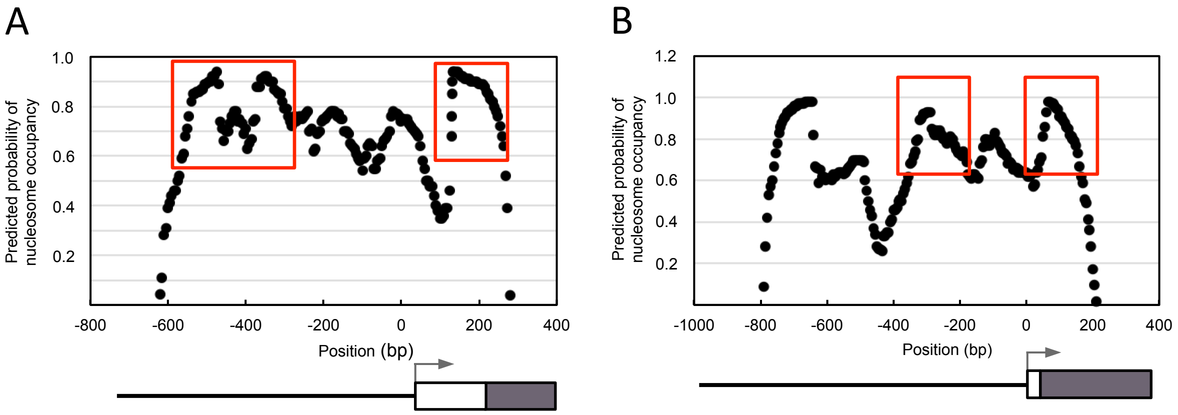
A
Predicted probability of nucleosome occupancy
1.0
0.8
0.6
0.4
0.2
-800
-600
-400
-200
0
200
400
Position (bp)
B
Predicted probability of nucleosome occupancy
1.2
1.0
0.8
0.6
0.4
0.2
-1000
-800
-600
-400
-200
0
200
400
Position (bp)

Figure S4

A

```
                          *        20         *        40         *        60         *        80         *       100         *
Bd_HTA1   : MAGKGGAKTTKAPVSRSSRAGLQFPVGRIHRQLKQRTQANRVGATAAVYSAAILEYLTAEVLELAGNASKDLKVKRITPRHLQLAIRGDEELDTLIKG-TIAGGVIPHIHKSLINK : 116
Bd_HTA2   : MSGRGKAKST-KVVSRSAKAGLQFPVGRIARYLKAGKYAERVGAGAPVYLSAVLEYLAAEVLELAGNAARDNKKNRIVPRHIQLAVRNDEELSKLLGTVTIANGGVLPKIHQVLLPK : 116
Bd_HTA3   : MAGRGKAIGSKKAISRSSKAGLQFPVGRIARFLKAGKYAERVGAGAPVYLAAVLEYLAAEVLELAGNAARDNKKTRIVPRHIQLAVRNDEELSRLLGMVTIASGGVMPNIHNLLLPK : 117
Bd_HTA4   : MAGRGKAIGAKKATSRSSKAGLQFPVGRIARFLKAGKYAERVGAGAPVYLAAVLEYLAAEVLELAGNAARDNKKTRIVPRHIQLAVRNDEELTKLLGGATIASGGVMPNIHQHLLPK : 117
Bd_HTA5   : MTPAGKGRKGKKAVTRSVKAGLQFPVGRVGRLLKKGRYAQRVGSGAPVYLAAVLEYLAAEVLELAGNAARDNKKSRIIPRHVLLAIRNDEELGKLLAGVTIAHGGVLPNIHTVLLPK : 117
Bd_HTA6   : MVGSKAGRRGKKPVSRSVKAGLQFPVGRIGRYLKLGRYAKRVGTGAPVYLAAVLEYLAAEVLELAGNAARDNKRNRIIPRHVLLAIRNDEELGKLLAGVTIAYGGVLPNINPVLLPK : 117
Bd_HTA7   : MGAGGKGKKGKKSVTRSVKAGLQFPVGRIGRFLKKGRYAQRVGSGAPVYLAAVLEYLAAEVLELAGNAARDNKKTRIIPRHVLLAIRNDEELGRLLGGVTIAHGGVLPNINPLLLPK : 117
Bd_HTA8   : ------GRKGKKAVSRSVKAGLTFPVGRIGRYLKHGRYAKRVGFGAPVFLAAVLEYLAAEVLELAGNAATDNKRKSIMPRHLLLAIRNDDELGKLLAGVTIAHGGVLPNIHSVLLPK : 111
Bd_HTA9   : MAGKGGAKTTKAPVSRSSRAGIQFPVGRIHRQLKQRVSANRVGATAAVYSAAILEYLTAEVLELAGNASKDLKVKRITPRHLQLAIRGDEELDTLIKG-TIAGGVIPHIHKSLINK : 116
Bd_HTA10  : MSGRGKVKGS-KAVSRSSKAGLQFPVGRIARYLKIGKYAQRVGAGAPVYLSAVLEYLAAEVLELAGNAARDNKKNRIVPRHIQLAVRNDEELSRLLGAVTIAAGGVLPNIHTTLLPK : 116
Bd_HTA11  : MGGKGGAKTAKLPVSRSSRAGLQFPVGRIHRQLKQRAQASRVGATAAVYSAAILEYLTAEVLELAGNASKDLKVKRITPRHLQLAIRGDEELDTLIKG-TIAGAGVIPHIHKSLINK : 116
Bd_HTA12  : MTGAGKGRKGKKSVTRSVKAGLQFPVGRIGRYLKKGRYAQRVGSGAPVYLAAVLEYLAAELLELAGNAARDNKKTRIIPRHLLLAIRNDDELGKLLAGVTIAHGGVMPKINQVLLPK : 117
At_HTA1   : MAGRGKTLGSKKATSRSSKAGLQFPVGRIARFLKAGKYAERVGAGAPVYLAAVLEYLAAEVLELAGNAARDNKKTRIVPRHIQLAVRNDEELSKLLGDVTIANGGVMPNIHNLLLPK : 117
At_HTA2   : MAGRGKQLGSKKSTSRSSKAGLQFPVGRIARFLKAGKYAERVGAGAPVYLAAVLEYLAAEVLELAGNAARDNKKTRIVPRHIQLAVRNDEELSKLLGDVTIANGGVMPNIHNLLLPK : 117
At_HTA3   : MSGRGKPKAT-KSVSRSSKAGLQFPVGRIARFLKAGKYAERVGAGAPVYLSAVLEYLAAEVLELAGNAARDNKKTRIVPRHIQLAVRNDEELSKLLGSVTIANGGVLPNIHQTLLPS : 116
At_HTA4   : ------CNTNKISAFENVREGEMFQVARIHKQLKNRVSAHSVGATDVVYMTSILEYLTTEVLQLAENTSKDLKVKRITPRHLQLAIRGDEELDTLIKG-TIIGGSVIPHIH------ : 104
At_HTA5   : MSGRGKPKAT-KSVSRSSKAGLQFPVGRIARFLKSGKYAERVGAGAPVYLSAVLEYLAAEVLELAGNAARDNKKTRIVPRHIQLAVRNDEELSKLLGSVTIANGGVLPNIHQTLLPS : 116
At_HTA6   : MS-TGKGRKGTKSVSKSMKAGLQFPVGRITRFLKKGRYAQRLGGGAPVYMAAVLEYLAAEVLELAGNAARDNKKSRIIPRHLLLAIRNDEELGKLLSGVTIAHGGVLPNINSVLLPK : 116
At_HTA7   : MSATTKGRKGKKSVSKSVKAGLQFPVGRIARYLKKGRYALRYGSGAPVYLAAVLEYLAAEVLELAGNAARDNKKNRINPRHLCLAIRNDEELGRLLHGVTIASGGVLPNINPVLLPK : 117
At_HTA8   : MAGKGGAKTTKKSISRSSRAGIQFPVGRIHRQLKQRVSAHRVGATAAVYTASILEYLTAEVLELAGNASKDLKVKRITPRHLQLAIRGDEELDTLIKG-TIAGGVIPHIHKSLVNK : 116
At_HTA9   : MSGKGAGKP-KKPITRSSRAGLQFPVGRVHRLLKTRSTAHRVGATAAVYTAAILEYLTAEVLELAGNASKDLKVKRISPRHLQLAIRGDEELDTLIKG-TIAGGVIPHIHKSLINK : 115
At_HTA10  : MAGRGKTLGSKKATTRSSKAGLQFPVGRIARFLKKGKYAERVGAGAPVYLAAVLEYLAAEVLELAGNAARDNKKTRIVPRHIQLAVRNDEELSKLLGDVTIANGGVMPNIHNLLLPK : 117
At_HTA11  : MAGKGGAKTMKKPISRSARAGIQFPVGRIHRQLKTRVSAHRVGATAAVYTASILEYLTAEVLELAGNASKDLKVKRITPRHLQLAIRGDEELDTLIKG-TIAGGVIPHIHKSLINK : 116
At_HTA12  : MSGTKVRRGGKKPVSRSVKSGLQFPVGRIGRYLKKGRYSKRVGTGAPVYLAAVLEYLAAEVLELAGNAARDNKKNRIIPRHVLLAVRNDEELGTLLKGVTIAHGGVLPNINPILLPK : 117
At_HTA13  : MAGRGKTLGSKKSTSRSSKAGLQFPVGRIARFLKNGKYATRVGAGAPVYLAAVLEYLAAEVLELAGNAARDNKKTRIVPRHIQLAVRNDEELSKLLGDVTIANGGVMPNIHSLLLPK : 117
Sc_H2A    : MSG-GKGGKA-AAKSRSAKAGLTFPVGRVHRLLRRGNYAQRIGSGAPVYLTAVLEYLAAEILELAGNAARDNKKTRIIPRHLQLAIRNDDELNKLLGNVTIAQGGVLPNIHQNLLPK : 115
Sc_Htz1   : MSGKGG----SGAKSSSARAGLQFPVGRIKRYLKRHATGRRVGSKAAIYLTAVLEYLTAEVLELAGNAAKDLKVKRITPRHLQLAIRGDDELDSLIRA-TIASGGVLPHINKALLLK : 112
Hs_H2A.Z  : MA--GG----TKAVSRSQRAGLQFPVGRIHRHLKSRTTSHRVGATAAVYSAAILEYLTAEVLELAGNASKDLKVKRITPRHLQLAIRGDEELDSLIKA-TIAGGVIPHIHKSLIGK : 110
Hs_H2A    : MSGRGKGGKA-KAKTRSSRAGLQFPVGRVHRLLRKGNYAERVGAGAPVYLAAVLEYLTAEILELAGNAARDNKKTRIIPRHLQLAIRNDEELNKLLGKVTIAQGGVLPNIQAVLLPK : 116
Hs_H2A.X  : MSGRGKGGKA-KAKSRSSRAGLQFPVGRVHRLLRKGHYAERVGAGAPVYLAAVLEYLTAEILELAGNAARDNKKTRIIPRHLQLAIRNDEELNKLLGGVTIAQGGVLPNIQAVLLPK : 116

              120
Bd_HTA1   : SSKE---- : 120
Bd_HTA2   : KAG-SQEF : 123
Bd_HTA3   : KAGGAAAD : 125
Bd_HTA4   : KASSIADD : 125
Bd_HTA5   : KTAESPKK : 125
Bd_HTA6   : KTAASPKK : 125
Bd_HTA7   : KAAESPKK : 125
Bd_HTA8   : RAAEKKTV : 119
Bd_HTA9   : TSKE---- : 120
Bd_HTA10  : KAGKSQEF : 124
Bd_HTA11  : SSKD---- : 120
Bd_HTA12  : KTAASPKK : 125
At_HTA1   : KAGAPQED : 125
At_HTA2   : KAGSTEED : 125
At_HTA3   : KVGKSQEF : 124
At_HTA4   : -------- :   -
At_HTA5   : KVGKSQEF : 124
At_HTA6   : KSATSPKK : 124
At_HTA7   : KSASSPKK : 125
At_HTA8   : VTKD---- : 120
At_HTA9   : SAKE---- : 119
At_HTA10  : KTGAAEDD : 125
At_HTA11  : TTKE---- : 120
At_HTA12  : KSEKSPKK : 125
At_HTA13  : KAGAADED : 125
Sc_H2A    : KSAKSQEL : 123
Sc_Htz1   : VEKK---- : 116
Hs_H2A.Z  : KGQQ---- : 114
Hs_H2A    : KTES---- : 120
Hs_H2A.X  : KTSKSQEY : 124
```

Figure S5

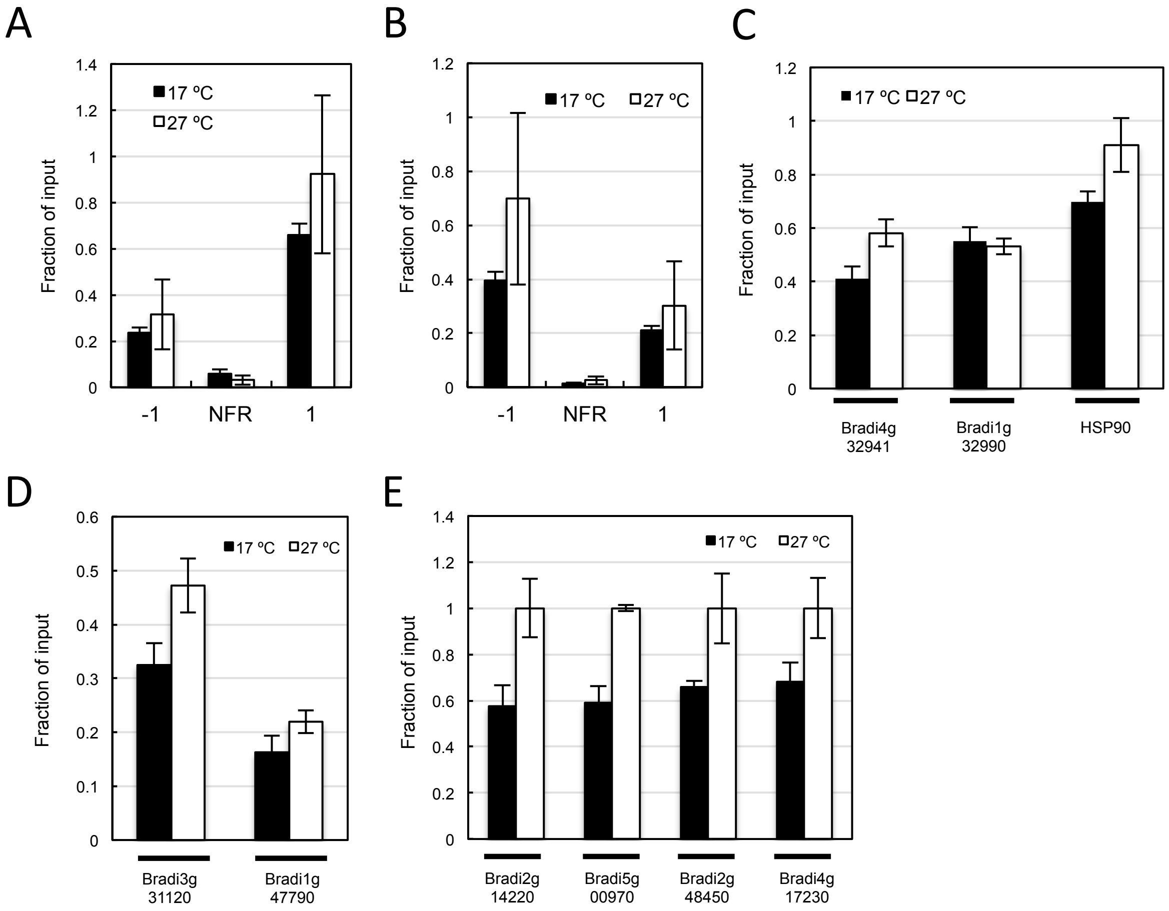
A
B
C
D
E
Fraction of input
17 ºC
27 ºC
-1
NFR
1
Bradi4g 32941
Bradi1g 32990
HSP90
Bradi3g 31120
Bradi1g 47790
Bradi2g 14220
Bradi5g 00970
Bradi2g 48450
Bradi4g 17230

Figure S6

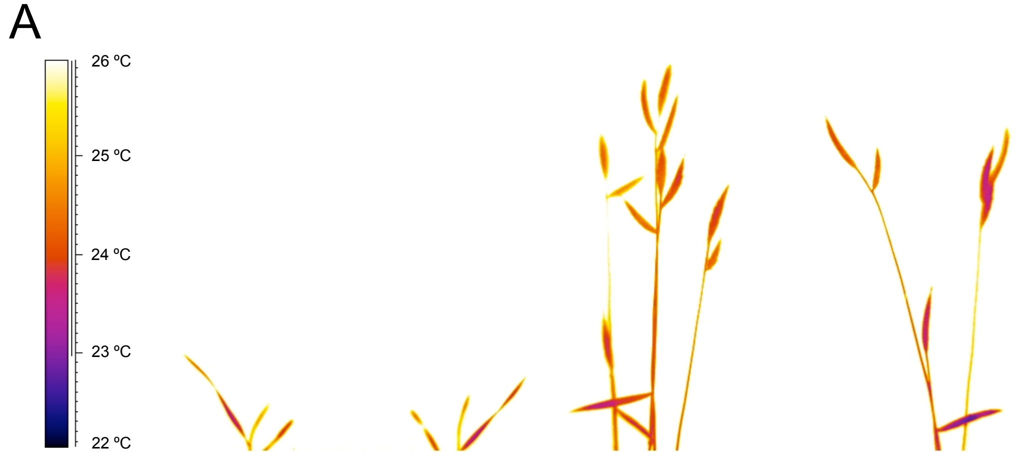

A
26 °C
25 °C
24 °C
23 °C
22 °C

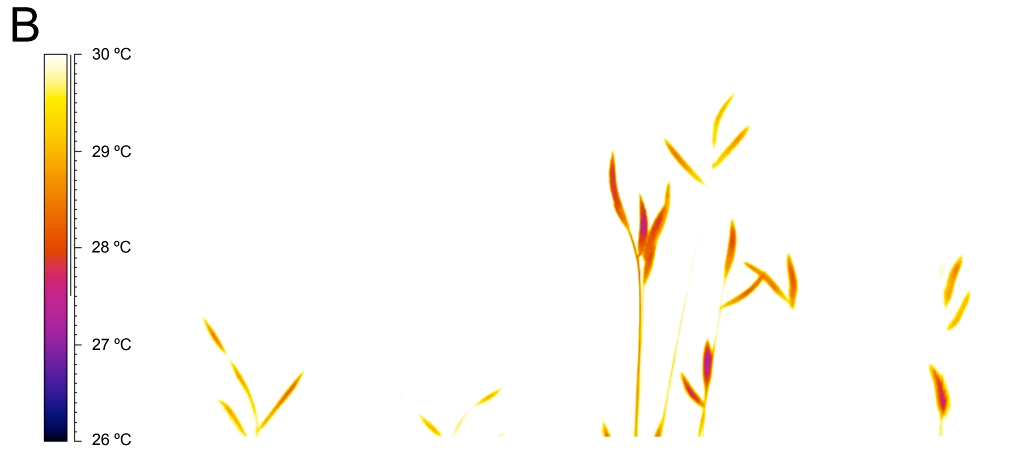

B
30 °C
29 °C
28 °C
27 °C
26 °C

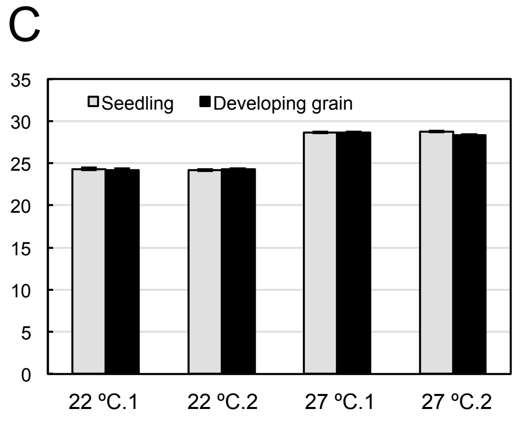

C
35
30
25
20
15
10
5
0
Seedling
Developing grain
22 °C.1
22 °C.2
27 °C.1
27 °C.2

Figure S7

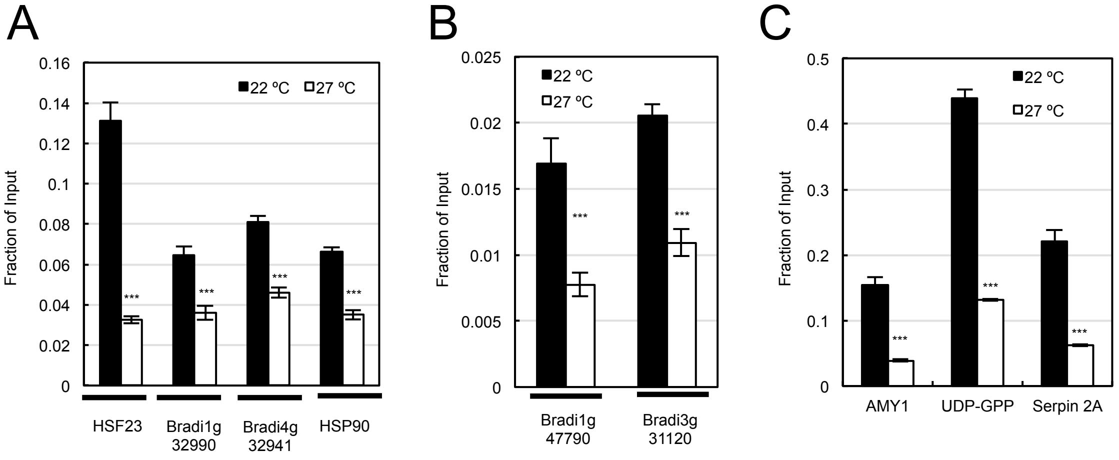
A
0.16
0.14
0.12
0.1
0.08
0.06
0.04
0.02
0
Fraction of Input
22 ºC
27 ºC
***
HSF23
Bradi1g 32990
Bradi4g 32941
HSP90
B
0.025
0.02
0.015
0.01
0.005
0
Fraction of Input
22 ºC
27 ºC
***
Bradi1g 47790
Bradi3g 31120
C
0.5
0.4
0.3
0.2
0.1
0
Fraction of Input
22 ºC
27 ºC
***
AMY1
UDP-GPP
Serpin 2A

Figure S8

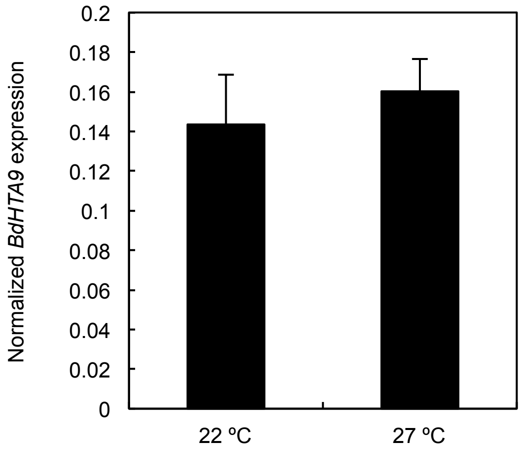

Normalized BdHTA9 expression
0.2
0.18
0.16
0.14
0.12
0.1
0.08
0.06
0.04
0.02
0
22 ºC
27 ºC

Figure S9

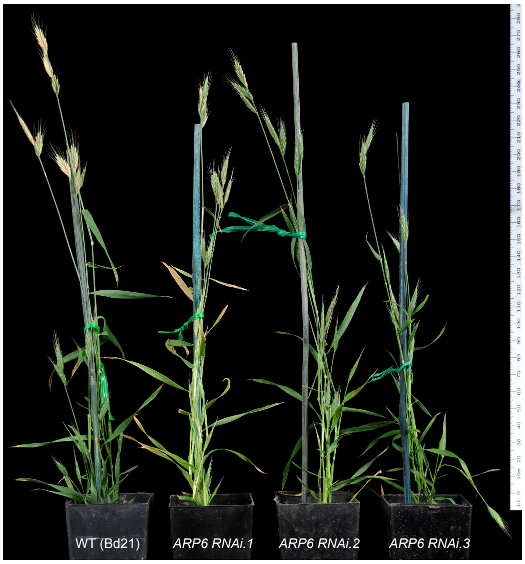

Table S1

Genotype	Number of florets/ spikelet	Percentage Infertile Florets
WT (Bd21) - 22 °C	7	12.5%
WT (Bd21) - 27 °C	6.9	26.3%
ARP6 RNAi.1	6	29.4%
ARP6 RNAi.2	6.5	27.8%
ARP6 RNAi.3	7	16.7%
