# Supplementary material for: Thermal stress effects on grain yield in Brachypodium distachyon occur via H2A.Z-nucleosomes
Source: Genome Biol. 2013 Jun 25;14(6):R65. doi: 10.1186/gb-2013-14-6-r65 (PMC4062847; doi:10.1186/gb-2013-14-6-r65)
Supplement: Additional file 4 — Table S3. [file gb-2013-14-6-r65-S4.DOCX]

**Table S3: Oligonucleotides used for chromatin immunopurification assays**

Oligonucleotide sequences used for *HSP70* nucleosome mapping/ChIP assay

| Amplicon | Sense | Anti-sense |
| --- | --- | --- |
| -528 | GTAAACCGCTCTAACCGCTC | GATTTACATCTGAAATCCGTAATAC |
| -478 | CGTCTTGGGTTGATTTATCTG | AGAAGTTCGTATAAATGTCTCCC |
| -420 | CTTCAGAGGCTTATGCGTCTC | AGTTACCGCCTTACGGGAG |
| -353 | CCCATTTTCCTCCCGTAAG | CTGCTTTTGAACGGTCTCTC |
| -309 | CAAGCTCTCGTGCACACTGTA | ACAACCAGGAGGCGACAC |
| -266 | CGTTCAAAAGCAGCCATGT | TCTGGAAGGGTCGGGAGT |
| -100 | GTTAAAACCCCTGCCGTCTC | TGGAAAGAGCTGTTTTGCTTG |
| -55 | CGATTTCCAGAGCAAGATTTC | CCTCCTCCTCTGCTACTCGTG |
| 20 | GTGCACGAGTAGCAGAGGAGG | CACCCCGACGCAGGAGTAG |
| 81 | TCGACCTGGGGACGACCTA | GACGTAGGAGGGCGTGGTC |
| 136 | AGATCATCGCCAACGACC | ATGGCGACCTGGTTCTTG |
| 185 | GCCTTCACCGACACCGAG | AGCAAGGAGGGATGGACAG |
|  | | |

Oligonucleotide sequences used for *HSF23* nucleosome mapping/ChIP assay

| Amplicon | Sense | Antisense |
| --- | --- | --- |
| -485 | CGGCGAGGAAGAGAATAAT | CGCTAAACCCTCTTACCCTAG |
| -453 | GTTTTGCCCCTAGTGCCG | CTCTACCCCCTCGATCCTTG |
| -369 | GGGGTAGAGGCGACCTTC | AGGTTTACGGCCAGGAGATC |
| -314 | CTTCCATGTCGCCGTCAAC | AGTGGGTTAGATAGATCCAAGGG |
| -222 | TCTAACCCACTATGGTGTCCAC | CCATAGGCCCAACTAAACTTC |
| -182 | GAAGATCAGAGCCCGAGAGG | TCCAGTCAGTTCAGTATCAAGCA |
| -68 | CGTGGTACTGGTTTTCGATC | TGCAGTCAGAACATTCCAGAAG |
| -21 | AAACCGACGTTGGTCTTCCC | AGGAAGAGGCCGACATAAAGC |
| 48 | CAGTGGAGATCGCAGCTTTATG | AAAGAAAACTGACGCAGATGAGC |
| 104 | AGTTCCGTCGCCGATATAAATC | TCTTTCTCTACGGGCGTGTTC |
| 140 | CTGCGTCAGTTTTCTTTCACC | CATCGAAGCCATTAGCACAA |
| 192 | CACGCCCGTAGAGAAAGAAGAC | CGCCAGGAGCTTCTCCATG |

Oligonucleotide sequences used for nucleosome mapping/ChIP assay of genes in developing endosperm.

| Locus ID | Gene | Oligonucleotides (Sense and Antisense) |
| --- | --- | --- |
| Bradi1g25440 | AMY1 | CGCAGTGTTTCCAAAGCATA  AGGGAGCATGACGTTGACTT |
| Bradi4g37350 | UDP-GPP | CCGACACGAAGATCGAGAAG  GGAGGCAGCAGAGAGAGAGA |
| Bradi1g14730 | Serpin 2A | GCACTCCGACGAACTACCA  GTTGGAGGGGGTGGATATG |

Oligonucleotide sequences used for nucleosome mapping/ChIP assay of genes that were up-regulated, down-regulated or displayed constant expression upon temperature shift.

| Locus ID | Amplicon | Oligonucleotides (Sense and Antisense) |
| --- | --- | --- |
| Bradi4g32941 | 16 | CCCTCTTATCCCTTTCGTCTC |
|  |  | AGTATGAGAGACACGGTACTGAGTT |
| Bradi4g32941 | 68 | CAGAACCACCAAACGATCAA |
|  |  | CCCAGGGTCCTGCTCAAC |
| Bradi1g32990 | 40 | GCTTCCACCTCCTTCGAC |
|  |  | TCTCTCTCCCCTCCCTCTCT |
| HSP90 | 85 | TATTCAACCACCGCATCG |
|  |  | TCGGCTCTCAACTAACTCTCA |
| HSP90 | 146 | TTTCACTTGAGAGTTAGTTGAGAGC |
|  |  | GTTGATCTCCGCCTGGAAC |
| Bradi3g31120 | 46 | GACGCTGCATTCGCTAAAA |
|  |  | CAGGCGACGACCCTTAGGT |
| Bradi3g31120 | 167 | ACCTAAGGGTCGTCGCCT |
|  |  | CCTGGTAGGACGGGATCTTT |
| Bradi2g36760 | 51 | GAGAGAGCACAACAGCAACAA |
|  |  | AATTCGGCAGCCTCCATG |
| Bradi2g14220 | 298 | CTGCCTACAGCCAACAACCT |
|  |  | GGTGGGGAGTAGACCGAGA |
| Bradi2g14220 | 63 | CATTCGCTCCTCCTCCTCT |
|  |  | TTATAGCCAGGCCCTTGTG |
| Bradi5g00970 | 60 | CAGCAATATAAATACACATCTAAGC |
|  |  | CTGCTTTCCTGTTAGGATTCG |
| Bradi5g00970 | 77 | CACAACATCACAACAAACAACAA |
|  |  | TAATTGCCAGGCCATGACT |
| Bradi2g48450 | -42 | GTGGATTTGAGTTGGGCATC |
|  |  | CCTCCTGCCATGTAATGTCTC |
| Bradi2g48450 | 3 | TCAATCGAATTGAGCCACAT |
|  |  | AGGAAGGAGACGACCAGCAC |
| Bradi4g17230 | 77 | TTAGCCACTGGTGAGCTAGGTA |
|  |  | CCCTCCTCACCTCCTCCAC |
| Bradi4g17230 | 132 | CAGCATCAGCTGTTTGTTCC |
|  |  | CGAGTAGCTCTACCTAGCTCACC |
