# Supplementary material for: Thermal stress effects on grain yield in Brachypodium distachyon occur via H2A.Z-nucleosomes
Source: Genome Biol. 2013 Jun 25;14(6):R65. doi: 10.1186/gb-2013-14-6-r65 (PMC4062847; doi:10.1186/gb-2013-14-6-r65)
Supplement: Additional file 5 — Table S4. [file gb-2013-14-6-r65-S5.DOCX]

Table S4: Gene identifiers of the HTA family in Brachypodium, Arabidopsis, human and yeast

| **Gene** | **Gene Locus ID** |
| --- | --- |
| BdHTA1 | Bradi1g09060 |
| BdHTA2 | Bradi1g10390 |
| BdHTA3 | Bradi1g25390 |
| BdHTA4 | Bradi1g25400 |
| BdHTA5 | Bradi1g66360 |
| BdHTA6 | Bradi2g23090 |
| BdHTA7 | Bradi2g37327 |
| BdHTA8 | Bradi2g62450 |
| BdHTA9 | Bradi3g26880 |
| BdHTA10 | Bradi4g06010 |
| BdHTA11 | Bradi4g14957 |
| BdHTA12 | Bradi4g28560 |
| AtHTA1 | At5g54640 |
| AtHTA2 | At4g27230 |
| AtHTA3 | At1g54690 |
| AtHTA4 | At4g13570 |
| AtHTA5 | At1g08880 |
| AtHTA6 | At5g59870 |
| AtHTA7 | At5g27670 |
| AtHTA8 | At2g38810 |
| AtHTA9 | At1g52740 |
| AtHTA10 | At1g51060 |
| AtHTA11 | At3g54560 |
| AtHTA12 | At5g02560 |
| AtHTA13 | At3g20670 |
| ScH2A | YDR225W |
| ScHtz1 | YOL012C |
| HsH2A.X | ENSG00000188486 |
| HsH2A | ENSG00000184348 |
| HsH2A.Z | ENSG00000164032 |
