# Supplementary material for: Thermal stress effects on grain yield in Brachypodium distachyon occur via H2A.Z-nucleosomes
Source: Genome Biol. 2013 Jun 25;14(6):R65. doi: 10.1186/gb-2013-14-6-r65 (PMC4062847; doi:10.1186/gb-2013-14-6-r65)
Supplement: Additional file 6 — Supplementary methods and figure legends. [file gb-2013-14-6-r65-S6.DOCX]

**SUPPLEMENTARY INFORMATION**

**METHODS**

**Thermal Imaging**

Thermal imaging was performed on plants grown at 22 and 27 ºC, at ZT 5 h. Thermal images were obtained using an FLIRSC660 thermal camera (FLIR) and analyzed using ThermaCAM Researcher Pro 2-10 (FLIR) software.

**FIGURE LEGENDS**

**Figure S1.** Expression of *Flowering Locus T* is promoted when Brachypodium is transferred from SD (14 h/10 h) to LD (20 h/4 h) photoperiod for 2 d. Samples were collected at ZT 19 h. Values are the mean of 3 biological replicates ± SE. (**, *P* < 0.01)

**Figure S2**. The Brachypodium transcriptome of vegetative seedlings responds to changes in ambient temperature. (*A-B*) Venn diagrams of total numbers of up-regulated (*A*) or down-regulated (*B*) DEGs in vegetative seedlings after 2 h shift to either 22 °C (green), 27 °C (red), or in both temperatures (yellow). DEGs with at least two-fold change in any of the temperature treatments were determined by two-way ANOVA (*P* for Temperature effect ≤ 0.05). The two-tailed *p*-values for the significance of the overlap represented under the Venn diagrams have been calculated using Fisher’s exact test. (*C-E*) Quantitative real-time PCR (qRT-PCR) analysis of genes that are up-regulated by increased temperature (*C*), down-regulated (*D*) or show constant expression (*E*) in vegetative seedlings 2 h after temperature-shift. Values are mean ± SE of 3 biological replicates.

**Figure S3**. Predicted nucleosome positions surrounding TSS of *HSF23* (*A*) and *HSP70* (*B*). Graphs display output of software prediction algorithm, with values presented for every five base pairs. In each schematic, the promoter (solid line), 5′UTR (white box), exons (black box) and TSS (arrow) are shown. Red rectangles indicate -1 and +1 nucleosomes as confirmed by H3 ChIP analysis. The transcription start site is at position 0 bp.

**Figure S4.** Multiple sequence alignment of Brachypodium, Arabidopsis, human and yeast HTAs proteins. HTA amino acid sequences were aligned using webPrank and the conserved part of the alignment was used for the construction of the phylogenetic tree. Different degrees of amino acid conservation are represented as different degrees of shade intensity using GeneDoc.

**Figure S5.** Nuclesome occupancy does not decrease upon shift to higher temperature in vegetative seedlings. (*A-C*) qRT-PCR analysis of MNase digested DNA from vegetative seedlings grown at 22 ºC or 27 ºC. Comparative occupancy is shown for -1 and +1 nucleosomes and nucleosome free regions (NFR) of *HSF23* (*A*) and *HSP70* (*B*) as well as +1 nucleosomes of genes whose expression was up-regulated (*C*), remained constant (*D*) or down-regulated (*E*) upon an increase in temperature. Values are mean ± SE of 3 biological replicates.

**Figure S6**. Brachypodium seedlings and emerged spikes have the same temperature in each growth condition. (*A*) Infra-red thermal images of seedlings (left) and emerged spikes (right) for two plants grown at 22 ºC. (*B*) Infra-red thermal images of seedlings (left) and emerged spikes (right) for two plants grown at 27 ºC. (*C*) Graph representing measurements of temperature taken from both seedlings and emerged spikes at each temperature. In *A* and *B*, colours on the thermal panel indicate the corresponding temperature measured for the plants in the respective conditions. Data are from 4 biological replicates, with 9-12 points recorded for each replicate. Values are the mean ± SE.

**Figure S7.** Nuclesome occupancy does decrease upon shift to higher temperature in developing grains. (*A-C*) qRT-PCR analysis of MNase digested DNA from vegetative seedlings grown at 22/17 ºC or 27/22 ºC. Comparative occupancy is shown for +1 nucleosomes of genes whose expression was up-regulated (*A*), remained constant (*B*) upon an increase in temperature, or have important roles in grain development (*C*). Values are mean ± SE of 3 biological replicates.

**Figure S8**. Normalized expression of *BdHTA9* in developing grain at 22/ 17 ºC and after 24 h treatment at 27/ 22 ºC. Grain were harvested at 8 DAP. Values are the mean of 3 biological replicates ± SE.

**Figure S9.** *ARP6 RNAi* lines do not display abnormal whole plant phenotypes. An image showing a representative plant of each *ARP6 RNAi* transgenic line used in this study and a wild-type (Bd21) plant grown in LD conditions at 22/ 17 ºC. Scale is indicated on the right side of the image.

**Table S1**. *ARP6 RNAi* lines display increased grain sterility. The number of florets without viable grain in the *ARP6 RNAi* lines were greater than wild-type Bd21 plants grown at the same temperature (22/ 17 ºC) and comparable to wild-type Bd21 plants grown at 27/ 22 ºC.
